# Oxidant-induced epithelial alarmin pathway mediates lung inflammation and functional decline following ultrafine carbon and ozone inhalation co-exposure

**DOI:** 10.1016/j.redox.2021.102092

**Published:** 2021-08-05

**Authors:** Nairrita Majumder, William T. Goldsmith, Vamsi K. Kodali, Murugesan Velayutham, Sherri A. Friend, Valery V. Khramtsov, Timothy R. Nurkiewicz, Aaron Erdely, Patti C. Zeidler-Erdely, Vince Castranova, Jack R. Harkema, Eric E. Kelley, Salik Hussain

**Affiliations:** a Department of Physiology and Pharmacology, School of Medicine, West Virginia University, USA; b Center for Inhalation Toxicology (iTOX), School of Medicine, West Virginia University, USA; c National Institute for Occupational Safety and Health, USA; d Department of Biochemistry, School of Medicine, West Virginia University, USA; e Department of Pathobiology and Diagnostic Investigation, School of Veterinary Medicine, Michigan State University, USA

**Keywords:** EPR, Free radical, Ozone, Carbon black, Inhalation Co-exposure, Lung inflammation

## Abstract

Environmental inhalation exposures are inherently mixed (gases and particles), yet regulations are still based on single toxicant exposures. While the impacts of individual components of environmental pollution have received substantial attention, the impact of inhalation co-exposures is poorly understood. Here, we mechanistically investigated pulmonary inflammation and lung function decline after inhalation co-exposure and individual exposures to ozone (O_3_) and ultrafine carbon black (CB). Environmentally/occupationally relevant lung deposition levels in mice were achieved after inhalation of stable aerosols with similar aerodynamic and mass median distributions. X-ray photoemission spectroscopy detected increased surface oxygen contents on particles in co-exposure aerosols. Compared with individual exposures, co-exposure aerosols produced greater acellular and cellular oxidants detected by electron paramagnetic resonance (EPR) spectroscopy, and *in vivo* immune-spin trapping (IST), as well as synergistically increased lavage neutrophils, lavage proteins and inflammation related gene/protein expression. Co-exposure induced a significantly greater respiratory function decline compared to individual exposure. A synthetic catalase-superoxide dismutase mimetic (EUK-134) significantly blunted lung inflammation and respiratory function decline confirming the role of oxidant imbalance. We identified a significant induction of epithelial alarmin (thymic stromal lymphopoietin-TSLP)-dependent interleukin-13 pathway after co-exposure, associated with increased mucin and interferon gene expression. We provided evidence of interactive outcomes after air pollution constituent co-exposure and identified a key mechanistic pathway that can potentially explain epidemiological observation of lung function decline after an acute peak of air pollution. Developing and studying the co-exposure scenario in a standardized and controlled fashion will enable a better mechanistic understanding of how environmental exposures result in adverse outcomes.

## Highlights

• Interaction with O_3_ mediates free radical production on the surface of carbon black (CB) particles.
• Oxidants mediate co-exposure (CB + O_3_)-induced lung function decline.
• EUK-134 (a synthetic superoxide-catalase mimetic) abrogates CB + O_3_-induced lung inflammation.
• CB + O_3_ co-exposure induces greater lung inflammation than individual exposures.
• Epithelial alarmin (TSLP) contributes significantly to the CB + O_3_ toxicity.

## Graphical abstract

**Figure:**
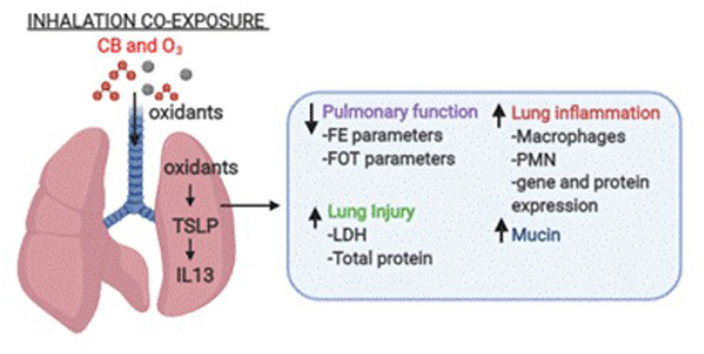

## 1 Introduction

The World Health Organization estimates that air pollution, a mixed exposure, is among the five leading causes of global morbidity and mortality. Although the overall mass burden of particulate pollutants has dropped over the last 20 years, due to tighter mass-based regulations and engineering advances, the incidences of lung and systemic pathologies are still on the rise. Almost 150 million individuals in the US breathe unhealthy levels of air pollution especially ultrafine particles (particles with aerodynamic diameter of 100 nm or less) and ground-level ozone (O_3_), which can induce and exacerbate lung injury [1]. Since the six cities study, demonstrating significant association of particulate matter with mortality, nearly all the scientific focus has been to study individual components of air pollution (e.g. particles, gases, etc.) [2]. While the impacts of individual components of environmental pollution have gained substantial attention, the impact of realistic co-exposures is poorly understood, even though, one inhales a mixture of gases and particles carrying a variety of products from anthropogenic activities [3]. Moreover, ultrafine particulate matter itself is a heterogenous mixture of different components: however, it is regulated based on mass irrespective of chemical constituents. The single toxicant mass based regulatory perspective of air pollution ignores the interactions between different constituents. These interactions may span from synergistic to antagonistic, and in our opinion, may very well be the basis of unexplained susceptibility/sensitivity to air pollution affirming the critical need to identify the mechanisms driving co-exposure toxicities. In the absence of such knowledge on environmental co-exposures, the development of effective and realistic exposure limits to safeguard public health will remain challenging, if not impossible.

Epidemiological data provide evidence of a sharp increase in adverse cardiorespiratory outcomes and hospitalizations after short term increases in air pollution [[4], [5], [6]]. However, the mechanisms driving this process remain undefined. Thymic stromal lymphopoietin (TSLP), an epithelial alarmin, has recently been demonstrated to have significant pathophysiological roles in a number of pulmonary and systemic disorders such as allergic pulmonary inflammation, cancer and autoimmunity [7,8]. Alarmins are endogenous molecules that can act as danger signals recognized by the immune system. A variety of immune and structural cell types such as dendritic cells, B and T lymphocyte subsets, mast cells, basophils, eosinophils and nature killer cells respond to TSLP stimulation to orchestrate a type 2 dominant immune response, a hallmark of chronic inflammatory disorders [8]. To the best of our knowledge, there is limited information on the role of TSLP in non-allergic environmental pollutant induced/modified inflammatory responses and lung function decline. Here, we investigated TSLP production and its role in inhalation co-exposure to ultrafine particles and gases.

Ground level O_3_, a criteria pollutant and toxicant of significant public health concern, is produced by the interaction of oxygen with volatile organic compounds in the presence of sunlight. It is a known trigger to exacerbate chronic lung diseases, and disproportionately impacts susceptible individuals, such as children, the elderly, and individuals with pre-existing inflammatory disorders [9]. The US Environmental Protection Agency (EPA) anticipates increased O_3_ levels over the next years due to climate change and anthropogenic activity [10]. Ultrafine carbon black (CB) is a known cause of pneumoconiosis and is considered possibly carcinogenic to humans (a Group 2B carcinogen) [11]. CB is a significant occupational/environmental exposure hazard with a global production of 8.1 million metric tons per year and a market value of $13 billion for applications [12]. Utilization of CB offers added advantage as it serves in a model system in which further contaminants can be added to study their specific impacts. Importantly, the high surface adsorption potential of CB particles creates an ideal carrier for other environmental toxicants. The majority of the experimental evidence for O_3_ or CB-induced toxic effects comes from individual exposures. Exposure to a combination of particles with a highly reactive gas such as O_3_ can sensitize cells for greater toxicity from a relatively non-toxic trigger (e.g., CB particles). Individual and/or sequential exposure to particulates (particulate matter (PM), CB, diesel exhaust particles) and O_3_ may induce lung injury, irritation, lung function decline, and cardiovascular impairments [[13], [14], [15], [16], [17], [18], [19], [20]]. However, co-exposures have not been extensively studied. In the few published co-exposure reports, cardiovascular outcomes (heart rate variability, cardiac arrhythmia, mechanical decrements) were the main focus [[21], [22], [23], [24], [25]]. Previous insights on CB and O_3_ pulmonary co-exposure were gained either after intratracheal instillation of relatively high doses O_3_-interacted CB particles or after sequential exposures to O_3_ and PM [[26], [27], [28], [29]]. Taken together, these studies clearly demonstrate the premise of interactive biological outcomes and warrant further studies to reveal mechanisms underpinning the increased toxicity of O_3_ and particle inhalation co-exposures.

Here, utilizing a physiologically relevant inhalation exposure route, pulmonary responses were quantified to separate vs co-exposure to a gaseous and particulate constituent of environmental pollution. We used CB as a surrogate of the carbon core of particulate matter as it represents a pure carbon samples free of other containments. The initial use of CB and ozone was to generate and characterize particle and gas simultaneous exposures by inhalation to provide a foundation for subsequent more intricate mixed exposure studies. The results indicate increased lung inflammation and lung function decline mechanistically driven in part by acellular and cellular oxidant generation and induced epithelial alarmin signaling.

## 2 Materials and Methods

**Exposure System and Aerosol Characterization:** An animal inhalation exposure system was designed to expose animals to either CB aerosols, O_3_ gas, or a mixture of the two toxicants (Supplemental Fig. S1). The design utilized a modified high-pressure acoustical generator (HPAG, IEStechno, Morgantown, WV) in which bulk CB material (Printex 90®, provided as a gift from Evonik, Frankfurt, Germany) generated ultrafine CB aerosols. The output from the HPAG was fed into a venturi pump (JS-60 M, Vaccon, Medway MA) to further deagglomerate particles. The real-time mass concentration (mg/m^3^) of aerosolized particles was monitored with a light scattering aerosol monitor (DataRAM, pDr-1500, Thermo Environmental Instruments Inc, Franklin, MA). O_3_ was produced by passing HEPA (High-efficiency particulate air) filtered dried air/pure oxygen through a corona discharge type O_3_ generator (HTU500AC, Ozone Solutions, Hull, IA). During co-exposures, the O_3_ was then mixed with the CB aerosol before entering the exposure chamber. O_3_ concentration in the chamber was measured using a calibrated O_3_ analyzer (Model 202, 2B Technologies, Inc., Boulder, CO). O_3_ monitor calibration was independently verified using Calorimetric ozone gas detector tubes (Sensidyne® LP, St Peterburgh FL). The O_3_ levels were maintained by adjusting the flow through the ozone generator based on the real-time readings from the ozone monitor.

Temperature and relative humidity in the exposure chamber were measured (HMT330, Vaisala, Helsinki, Finland) and maintained at 20–22 °C and 50–70% respectively. Exposure chamber and animal housing cages are made with Stainless steel (grade 316) which has excellent compatibility with O_3_. The whole-body stainless-steel exposure chamber (Cube 150, IEStechno, Morgantown, WV) individually housed up to 36 mice. Gravimetric measurements of the mass concentration were collected and reported for each exposure and were also used to continually calibrate the DataRAM. Particle size distributions were sampled from the exposure chamber with: 1) an electrical low-pressure impactor (ELPI+, Dakati, Tempera, Finland), 2) an aerosol particle sizer (APS 3321, TSI Inc Shoreview, MN), 3) a scanning mobility particle sizer (SMPS 3938, TSI Inc. Shoreview, MN), and 4) a Nano Micro-orifice Uniform Deposit Impactor (Moudi 115R, MSP Corp, Shoreview, MN). Aerosols were collected on formvar coated copper grids and imaged using JOEL 1400 transmission electron microscope (JOEL, Tokyo, Japan) to characterize morphology. Polycarbonate filters were used to collect the morphology characterization using a field-emission scanning electron microscope (Hitachi S4800, Tokyo, Japan). Elemental composition of particle surfaces was analyzed by X-Ray Photoelectron Spectroscopy (XPS) (Physical Electronics PHI 5000 VersaProbe XPS/UPS). After the aerosol/gas left the chamber it was HEPA and charcoal filtered before entering the house exhaust. Software was developed (IEStechno, Morgantown, WV) to monitor, control and record system parameters during exposures. Various feedback loops were utilized in the software to hold concentration and pressure levels constant during exposures. The pressure in the HPAG was held constant to improve aerosol generation. The chamber pressure was also held constant at zero for animal comfort and to minimize any potential chamber leaks. The DataRAM real-time values were utilized in a feedback loop to hold the aerosol concentration steady by changing the power delivered to the HPAG.

**Electron Paramagnetic Resonance (EPR) Spectroscopic Studies:** Purified 5,5-dimethyl-1-pyrroline-N-oxide (DMPO) was purchased from Dojindo laboratories, Kumamoto, Japan. Xanthine and xanthine oxidase (XO) from bovine milk (catalog number: X4875) were purchased from Sigma-Aldrich, USA. All the EPR spin trapping and spin probe experiments were carried out in phosphate buffered saline (PBS, pH7.4) pre-treated with Chelex. EPR spin trap experiments were performed using a spin trap DMPO. EPR spectra were recorded using a Bruker EMXnano spectrometer (Bruker BioSciences, Billerica, MA, USA) operating at X-band with a 100 kHz modulation frequency as described previously [30]. Data acquisition was performed using Bruker Xenon-nano software. Solid/powder samples were loaded directly in to an EPR quartz tube (O.D 4 mm). Liquid samples of 50 μL were loaded into glass capillary tubes that were sealed on one end using Critoseal clay and placed inside the 4 mm (O.D.) EPR quartz tube. The quartz tube was positioned inside the resonator/cavity and EPR spectra were recorded at room temperature. The following settings were used: microwave frequency, 9.615 GHz; sweep width, 100 G (200 G for powder); microwave power, 20 mW; modulation amplitude, 0.5 G (5 G for powder); modulation frequency, 100 kHz; receiver gain, 60 dB; time constant, 41 ms (20.5 ms for powder); conversion time, 15 ms (31 ms for powder), sweep time, 30 s (50 s for powder); number of scans, 1 or 10.

**Ferric Reducing Ability of Serum (FRAS) Assay:** FRAS assay was performed to study acellular oxidant generation ability of individual and co-exposures by following the already published methodology [31,32] with a slight modification. In order to accurately mimic the inhalation exposure and eliminate the artifacts that might arise due to interaction with room air, we bubbled aerosols (air, CB, O_3_ and CB + O_3_) for 5 min through the human serum and proceeded to quantify the changes by exactly following the previously published standardized methodology. In order to validate the occurrence of interactions between O_3_ and CB at levels to which population is chronically exposed, we performed FRAS assay using two exposure concentrations for CB (250 μg/m^3^ (low dose) and 10 mg/m^3^ (high dose)) and O_3_ (200 ppb and 2 ppm).

**Animal Exposures and Exposure Conditions**: C57BL/6J male mice (8 weeks old) were purchased from Jackson Laboratory (Bar Harbor, ME) and acclimated at the West Virginia University Animal Care Facility before exposure. All animals were maintained in a room with a 12-h light/dark cycle and provided chow and water ad libitum. All the animal procedures were approved by the WVU Animal Care and Use Committee. Animals were exposed for 3 h per day, up to 2 days, to either filtered air, O_3_ (2 ppm), CB (10 mg/m^3^), or CB + O_3_ (10 mg/m^3^ + 2 ppm). EUK-134 (Cayman Chemicals, Ann Arbor, MI) is a catalase/SOD mimetic which prevents oxidative stress. EUK-134 (10 mg/kg) was intraperitoneally injected 30 min prior to exposure and mice were sacrificed 24 h post exposure. TSLP neutralizing antibody (catalog # MAB555-100) and TSLP iso-type antibody (catalog # MAB002) (R&D systems, MN) were administered (0.8 mg/kg) by nasal and oropharyngeal aspiration 1 h before exposure. Antibody administration was performed by both nasal and oropharyngeal routes (half dose by either route) to maximize neutralization of TSLP in both upper and lower airways. TSLP-isotype antibody serves as more specific control for TSLP neutralizing antibody thus eliminating the need of doing PBS only group. The mice were euthanized by intraperitoneal injection of Fatal Plus (250 mg/kg) and analyzed 24 h following the exposure. Details on animal cohorts used for different experiments is presented in Supplemental Table S1. Schematics for exposures are presented in Supplemental Fig. S2.

**Carbon Black Lung Burden Quantification:** Lung burden was quantified according to a previously described method with slight modifications [33]. A group of animals (5–7) were euthanized within 15 min of inhalation exposure, lungs were removed, and wet lung weight was quantified. Lung tissue was minced and digested in a 25% KOH/methanol (w/v) solution at 60 °C overnight in a dry heating block. After digestion tubes containing lung samples were vortexed and centrifuged at 16,0000 g for 10 min at 25 °C. Pellet was resuspended in 50% HNO_3_/Methanol (v/v) and incubated at 60 °C for 3 h in heating block. Tubes containing lung samples were vortexed and centrifuged at 16,0000 g for 10 min at 25 °C. Pelleted samples and known standards of CB and O_3_ interacted CB collected from inhalation chamber were resuspended in surfactant water solution (10% NP-40). Samples and standards (1 mg/mL- 1.56 μg/mL) were spectrophotometrically read at 690 nm, sonicated and re-read till a stable optical density was obtained [33].

**Bronchoalveolar Lavage Fluid (BALF) Collection and Analyses:** Whole lung bronchoalveolar lavage (BAL) was performed to collect 3 mL of BALF (3 washes of 1 ml each), pooled and processed for cellular and biochemical analyses as described previously [34,35]. BALF total cells were quantified using a hemocytometer/automated cell counter (Countess®, Thermofisher Scientific, Waltham, MA). Differential cell counts were performed after cytospin preparation (Cytospin® Thermofisher Scientific, Waltham, MA) as described by us previously [34]. Cells were stained in Hema3 (Fisher Scientific, Pittsburgh, PA). Percentage of different cells types (macrophages, neutrophils, lymphocytes, eosinophils etc.) were calculated and absolute cell numbers were determined by taking into consideration the volume of lavage fluid collected. Lavage proteins were quantified as a marker for air-blood barrier integrity by Pierce BCA kit (Thermofisher Scientific, Waltham, MA) according to manufacturer's instructions. Lung cell death was estimated by quantifying lactate dehydrogenase (LDH) activity by Cytotox 96 NonRadioactive Cytotoxicity Assay (Promega, Madison, WI) according to manufacturer's instructions and previously published reports [35].

***In Vivo* Immunospin Trapping (IST):** IST employs antibody-based detection of stable adducts formed by the reaction of free radicals with a spin trap. IST was performed following the methods published by us previously [36]. Briefly, mice were intraperitoneally injected with 5,5-dimethyl-1-pyrroline-N-oxide (DMPO) 24, 18 and 12 h before exposure (0.5 g/kg for each injection and thus 1.5 g/kg total dose). Mice were exposed by inhalation to filtered air or 10 mg/m^3^ CB + 2 ppm O_3_ co-exposure aerosols for 3 h and sacrificed 24 h post exposure. Lung tissue were immunostained for epithelial cells (EPCAM monoclonal antibody G8.8, Developmental Studies Hybridoma Bank, University of Iowa), actin (Phalloidin, Thermofisher Scientific, Waltham, MA) and nuclei (DAPI, Thermofisher Scientific, Waltham, MA). The rabbit polyclonal anti-DMPO antibody was a kind gift from Dr. Ron Mason (National Institute of Environmental Health Sciences, NIEHS).

**Enzyme Linked Immunosorbent Assay (ELISA)**: ELISA assays were performed for keratinocyte chemoattractant (KC), tumor necrosis factor-α (TNF-α), interleukin-6 (IL-6), interleukin-13 (IL-13), interleukin-1β (IL-1β) and thymic stromal lymphopoietin (TSLP) using Duoset sandwich ELISA assay kits (R&D Systems, MN) according to manufacturer's recommendations. Lower limit of detection for these assays were IL-1β (15.6 pg/mL), TNF-α (31.3 pg/mL), KC (15.6 pg/mL), IL-6 (15.6 pg/mL), IL-13 (62.5 pg/mL) and TSLP (15.6 pg/mL).

**Lung Histology**: Lungs were fixed with 10% neutral buffered formalin instillation through the trachea till fully distended. Hematoxylin and eosin staining was performed on 5 μm thick sections. Tissues were evaluated by a board-certified veterinary pathologist in a blinded fashion.

**Real-time PCR Gene Expression:** The lung tissues were snap frozen in liquid nitrogen for PCR analyses. Total RNA was extracted using Qiagen RNeasy RNA isolation kit (Qiagen, Germantown, MD) and cDNA was synthesized using Reverse Transcription Kit (High-Capacity cDNA Reverse Transcription Kit, Thermofisher Scientific). Sequences of PCR primers are provided in Supporting information Table S2. PCR reaction was performed in triplicate using AriaMX real time PCR machine (Agilent, Santa Clara CA) using syber green chemistry as described by us previously [37]. Relative expression level of genes of interest was measured using the comparative threshold method with 18S as internal control. Data were analyzed using ΔΔCt method, where fold change = 2^−ΔΔCt^.

**Lung Function Measurements:** Forced Oscillation technique (FOT) and forced expiration (FE) measurements were performed 24 h post exposure after exposure using FlexiVent mechanical ventilator system (SCIREQ, Inc., Montreal, Canada) equipped with FX1 module as well as negative pressure forced expiration (NPFE) extension. Data was captured and analyzed using flexiWare v7.2 software. Aerosol challenges to (0–100 mg/mL) methacholine (2s each) was performed using synchronized nebulizer activation (Aeroneb Lab nebulizer, 2.5–4 μm; Aerogen, Galway, Ireland) integrated in the inspiratory arm of the Y-tubing. Protocol for these measurements is already described in detail [38]. Briefly, mice were anesthetized with sodium pentobarbital (70 mg/kg) or urethane (2 mg/kg), a metal tracheal cannula (18 gauge, 0.3 cmH_2_O.s/mL resistance) was inserted. Quasi-sinusoidally ventilation with a tidal volume of 10 mL/kg, a frequency of 150 breaths/min, an inspiratory to expiratory ratio of 2:3, and a positive end-expiratory pressure of 3 cmH_2_O was performed. After two deep inflations (30 cmH_2_O pressure), baseline measurements were performed by applying a broadband forced oscillation waveform inducing frequencies between 0.5 and 19.75 Hz (Prime-8; P8) and were analyzed by the constant-phase model. Newtonian resistance (Rn, airway resistance) was inferred from this data. Overall resistive and elastic properties of the respiratory system were measured using a snapshot 150 perturbation which is a single frequency forced oscillation (matched to subject's ventilation frequency and tidal volume). Data from this Snapshot measurement was fitted to single compartment model and Respiratory system resistance (Rrs) and compliance (Crs) were calculated. Same perturbations were applied in conjunction with increasing doses of methacholine (0–100 mg/mL) to construct dose response [38,39]. During these measurements after performing snap shot measurements, a Quick Prime-3 (QP3) perturbation was applied for five runs at approximately 15 s apart, resulting in 5 measurements for each concentration of methacholine. Each sequence was followed by a NPFE measurement taken approximately 15 s after the last FOT measurement using NPFE extension for FlexiVent. Forced Expiratory Volume at 0.1 s (FEV0.1) was measured in triplicate for each dose of methacholine. Moreover, a provocative concentration 20 (PC20), inducing a 20% decrease in FEV0.1 was assessed, by calculating the slope of the dose-response curve of each individual mouse, where the peak responses to MCh were normalized to the FEV0.1 of 0 mg/ml MCh (=100%).

**Statistical Analyses:** Data are presented as means ± standard deviation (SD) from at least two repeats with a total of 5–10 animals per group. Depending on group size normality of the data was confirmed by suitable normality tests (D'Agostino-Pearson or Shapiro-Wilk). In case of normally distributed data, significant differences between groups were identified by analysis of variance (one-way or two-way, as dictated by experimental design) and Tukey's post hoc test was applied. If data failed normality test, a non-parametric testing was performed, and a Kruskal Wallis post-test for group differences was applied. Individual comparisons between groups were confirmed by Student-t test or Mann-Whitney *U* test as appropriate. For null hypothesis, a two-tailed p-value of less than 0.05 (95% confidence level) was considered statistically significant. Statistics were performed using GraphPad Prism v7.

## **3** Results

### 3.1 Exposure system and aerosol characterization

**Fig. 1:**
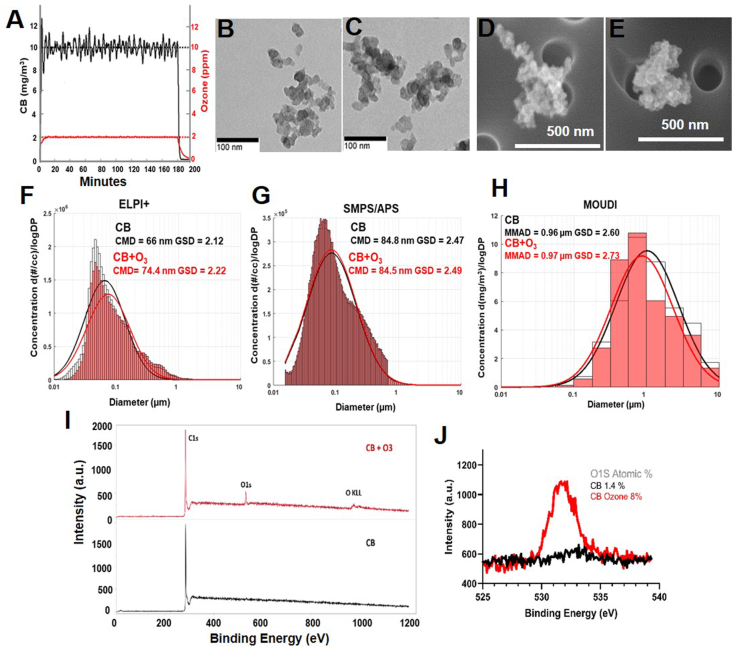
**Aerosol real time monitoring and characterization. A**) Real-time monitoring of CB and O_3_ exposure concentrations. Representative Transmission Electron Microscope images of **B**) CB, **C)** CB + O_3_ and Scanning Electron Microscope images of **D**) CB and **E**) CB + O_3_ aerosols collected from inhalation chamber. Aerosol size distribution measurements by **F**) Electrical low-pressure impactor (ELPI+), **G**) Scanning mobility particle sizer (SMPS)/Aerosol particle sizer (APS) and **H**) micro-orifice uniform deposit impactor (MOUDI). **I**) X-ray photo electron spectroscopy (XPS) spectral scan of CB and CB + O_3_ particles collected on filters after aerosolization. **J**) O1S peaks from XPS spectra.

Real-time monitoring of the aerosol and gas levels from the CB and CB + O_3_ co-exposures confirmed that we generated stable aerosols over the exposure period (Fig. 1A). The aerosol size distributions were characterized using a multitude of techniques (Fig. 1B–J). To visualize the morphology and characterize the aerosols TEM and SEM were performed on the aerosols collected from the exposure chamber after CB and CB + O_3_ exposure (Fig. 1B–E). Charge-based particle size measurements conducted with the ELPI + resulted in a count median diameter of 66 and 74.5 nm with geometric standard deviations of 2.13 and 2.20 for CB and CB + O_3_ respectively (Fig. 1F). Concurrent SMPS/APS measurements that covered the range of chamber aerosols indicated that a majority of particulates were in the nano/ultrafine size range and had a count median diameter of 84.8 and 84.5 nm with geometric standard deviations of 2.47 and 2.49, respectively for CB and CB + O_3_ (Fig. 1G). Mass-based Moudi measurements showed a mass median aerodynamic diameter of 0.96 and 0.97 μm with a geometric standard deviation of 2.60 and 2.73, respectively, for CB and CB + O_3_ (Fig. 1H). Both the aerosol types had irregular morphology and formed aggregates. CB only aerosols had a loose agglomerate structure compared to CB + O_3_ aerosols. The primary particulate that formed the agglomerates had a diameter of 18 ± 6 nm. Chemical modification on the surface of CB with and without O_3_ co-exposure was determined using X-ray photoelectron spectroscopy (XPS). The XPS survey scan (Fig. 1I) confirmed the presence of C1s peak with CB and presence of C1s, O1s, and O KLL with CB + O_3_. High resolution spectra of C1S, O1s and O KLL (Auger) showed unambiguous increase in O1S (Fig. 1J) with CB + O_3_ and not with CB, confirming oxidation of CB due to O_3_ co-exposure.

### 3.2 Interaction with ozone induces acellular oxidant generation on CB surface

**Fig. 2:**
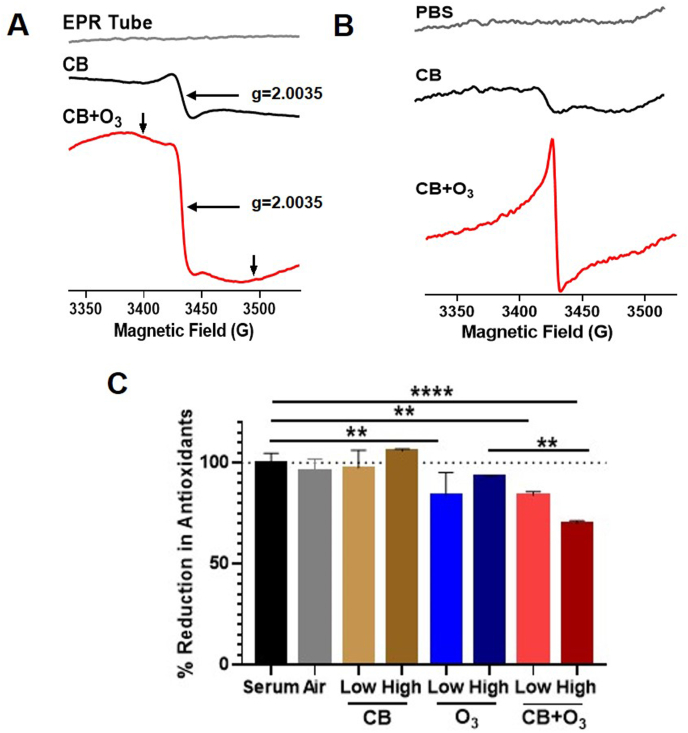
**Interaction with ozone significantly increase acellular oxidant potential of carbon black (CB) particles. A)** Electron paramagnetic resonance (EPR) spectra of CB particles and O_3_ treated CB particles. **B**) EPR spectra of CB particles and O_3_ treated CB particles in PBS. **C**) FRAS assay for acellular oxidant generation potential of single and co-exposure aerosols at low (250 μg/m^3^ CB and/or 200 ppb O_3_) and high (10 mg/m^3^ CB and/or 2 ppm O_3_) exposure dose. Data is presented as mean ± standard error of mean of 2 independent experiment with at quadruplicate of each condition at each time.

Changes in O_3_-induced CB surface reactivity, in terms of a cellular free radical generation and lung inflammation, were elaborated further. The EPR spectra of CB and O_3_-reacted CB powder are shown in Fig. 2A. A carbon center localized free radical demonstrated a strong EPR signal at g = 2.0035 in case of ultrafine CB. The signal intensity for this radical was greater in O_3_-reacted CB (even with half of the amount of material being used for assay). The increased signal intensity was due to the formation of new carbon centered radicals by the reaction between reactive/oxidizing ozone molecule and reactive carbon centers. In addition, a broad EPR signal, as marked by the arrows, was also observed in O_3_-reacted CB, which potentially stems from the trapped oxygen centers on the CB. To understand the reactive surfaces of the CB in aqueous medium EPR spectra were recorded for the CB particles suspended in the phosphate buffer solution (PBS) (Fig. 2 B). CB in PBS shows a weak signal. However, O_3_-treated CB in PBS shows a strong signal. No EPR signal was obtained without the particle. These results demonstrate that the carbon black particles retain the reactive surface area after exposure to aqueous medium. The reactivity arising from the interaction of CB with O_3_ was confirmed in a FRAS assay at two exposure concentrations, which demonstrates that, indeed, these reactive surfaces significantly decrease the quantity of antioxidants in the human serum and such interactions occur at even very low ambient levels (Fig. 2 C).

**Fig. 3:**
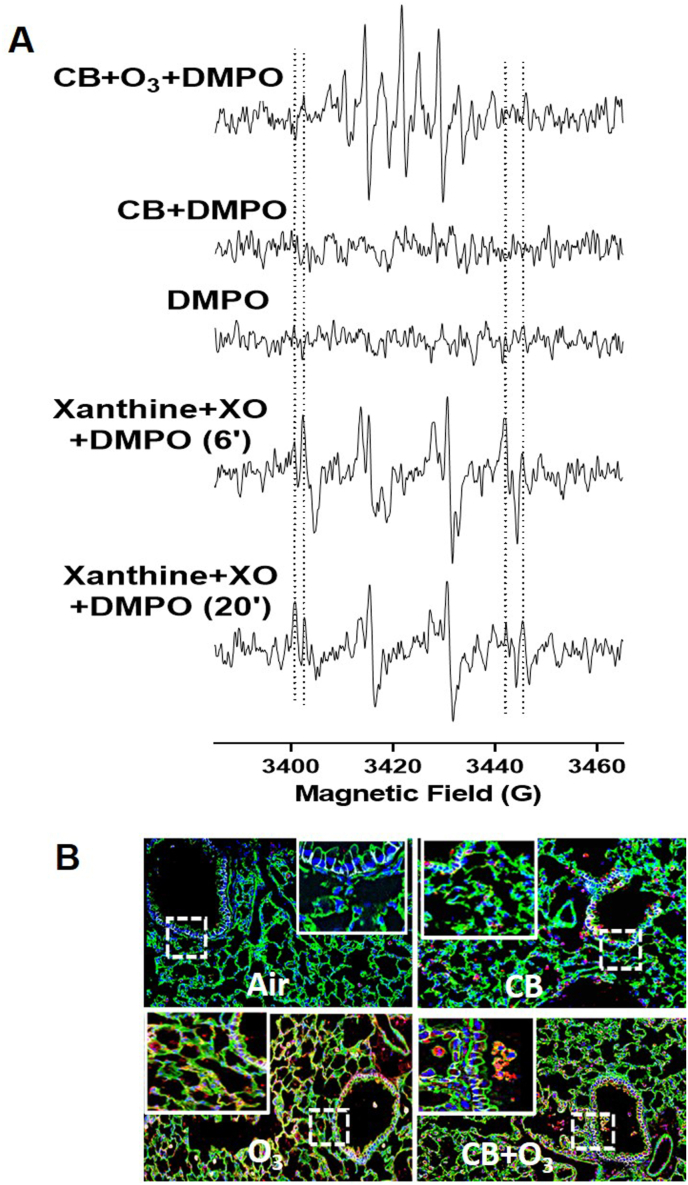
**Interaction with ozone significantly increase acellular and in vivo oxidant generation potential of CB particles**. **A**) EPR spectra of free radical adducts of DMPO. All the reactions were performed in PBS. Lines represent EPR spectra of DMPO (0.1 M) + O_3_ treated CB (1 mg), DMPO (0.1 M) + CB (1 mg), DMPO (0.1 M), DMPO (0.1 M) + XO (10 mU/mL) + Xanthine (0.2 mM). Spectra of DMPO (0.1 M) + XO (10 mU/mL) + Xanthine (0.2 mM) were recorded 6 min and 20 min after mixing the reactants. The low and high field EPR peaks of DMPO-OH and DMPO-OOH were marked with vertical dotted lines for easy visualization. EPR instrument parameters used were as described under Materials and Methods section. B**)** Representative immunospin trapping (IST) images of mice exposed to air, CB (10 mg/m^3^), O_3_ (2 ppm) or CB + O_3_ (10 mg/m^3^ CB and 2 ppm O_3_) for 3 h and sacrificed 24 h post exposure. The figure represents epithelial cells (EPCAM green), nuclei (DAPI, blue) and free radicals (DMPO-red). (For interpretation of the references to colour in this figure legend, the reader is referred to the Web version of this article.)

To investigate whether the CB particles can release soluble free radical(s) in PBS upon exposure to O_3_, EPR spin trapping experiments were carried out, using DMPO as a spin trap. The reaction mixture containing spin trap DMPO, CB or O_3_-treated CB in PBS are shown in Fig. 3 A. No EPR signal was observed from the CB sample. However, O_3_-treated CB in PBS shows an EPR spectrum with multiple peaks. To understand the type of free radicals produced by the O_3_-treated CB in PBS EPR spin trapping experiments were carried using known source of superoxide radical generating xanthine oxidase (XO) and xanthine system [40]. EPR spectra were recorded for the reaction mixture containing DMPO (0.1 M), XO (10 mU/mL), and xanthine (0.2 mM) in PBS. The corresponding EPR spectrum was recorded 5 min after mixing the reactants. EPR spectrum is dominated by the peaks corresponding to superoxide radical adducts of DMPO, DMPO-OOH. A weak signal corresponding to the hydroxyl radical adducts of DMPO, DMPO-OH, was also observed. The DMPO–OOH adduct decomposes to DMPO–OH in aqueous solution with a half-life of approximately 45 s [41,42]. Hence, we further recorded the EPR spectrum for the same sample after 20 min. Now the EPR spectrum was dominated by the strong signal hydroxyl radical adduct of DMPO, DMPO-OH strong and over the weak signal of superoxide radical adduct of DMPO, DMPO-OOH. These results demonstrates that the EPR signals shown by the O_3_-treated CB in PBS are not due to the DMPO-OOH and DMPO-OH radical adducts. Hence, the spin trap (DMPO) is directly oxidized by the radicals/reactive species on the surface of the O_3_-treated carbon particles to form the DMPO-X adducts as described previously [43,44]. Increased intracellular in vivo free radical production was confirmed by an in vivo immuno spin trapping (IST) assay (Fig. 3 B). IST of lung tissue confirmed increased oxidant production by both O_3_ and CB + O_3_ inhalation co-exposure.

### 3.3 Increased potency of CB and O_3_ inhalation Co-exposures

**Fig. 4:**
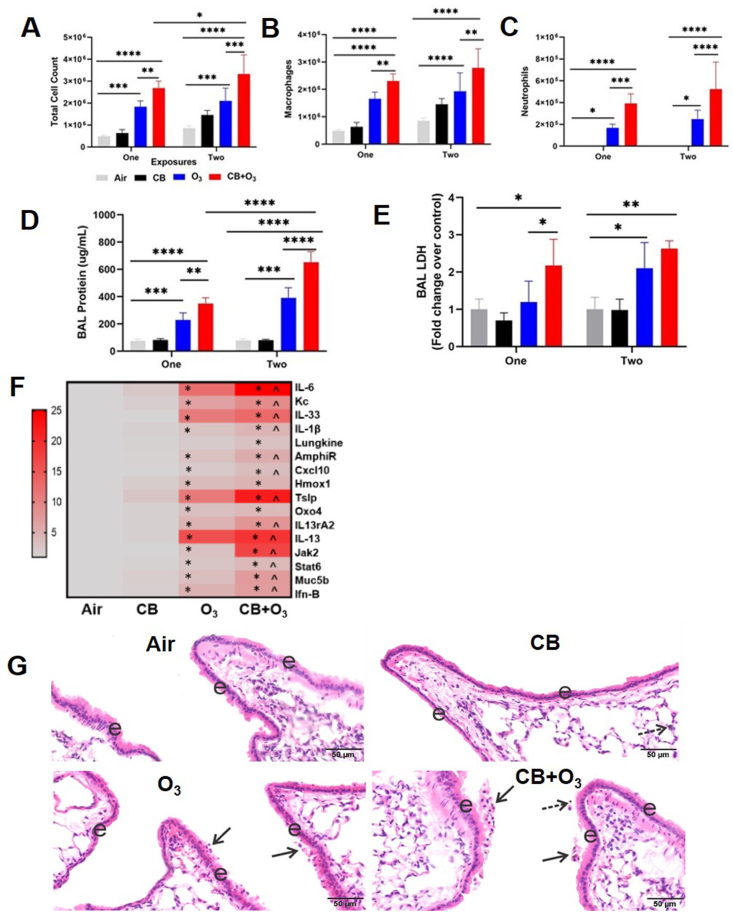
**Increased potency of co-exposures to induce lung inflammation.** Broncho-alveolar lavage **A**) total cells, **B**) macrophages, **C**) neutrophils, **D**) total proteins and **E**), Lactate Dehydrogenase (LDH) **F**) Lung tissue mRNA expression of inflammatory markers, **G**) H&E staining of lung tissue. “e” indicated epithelium and “arrow” indicate necrotic epithelial cells. Data are presented as mean ± SD of n = 5–7 mice per group and analyzed by two-way analysis of variance (ANOVA) followed by Tukey's post hoc test. PCR values are presented as fold change values. * represents significantly different from control while ^ presents significantly different between O_3_ and co-exposure group. *p < 0.05, *p < 0.01, ***p < 0.001.

To explore the relative biological activity of co-exposure aerosols compared with single exposure, mice were exposed either one or two times (24 h apart) to individual or co-exposure aerosols and lung inflammation and lung function decline were measured. Significantly greater numbers of total cells, macrophages and neutrophils in the BALF were observed after co-exposure compared with all other exposures (Fig. 4 A-C). These differences from control were more pronounced after two exposures. A similar significant increase in protein contents and lactate dehydrogenase (LDH) activity of the BALF indicated an increased permeability of air-blood barrier and lung cell death respectively (Fig. 4 D-E). An increase in pulmonary inflammation with co-exposures was observed, as seen from neutrophil influx, a marker for inflammation, and was further confirmed with real-time PCR analysis of the lung homogenate. PCR analysis further confirmed that co-exposure causes a greater pulmonary inflammatory response compared to individual exposures (Fig. 4F). Significant increases in mRNA expression for epithelial alarmins (TSLP, IL-33), inflammatory cytokines/chemokines (KC, IL-6, IL-1β, Lungkine, CXCL10), mucin (Muc5b), and IL-13 signaling pathway (IL-13rα, Jak 2, Stat-6, IL-13) was detected. Except for lungkine, Hmox, and Oxo40, co-exposure induced significantly greater mRNA expression compared with individual exposure groups. Concentration of some of these BALF cytokines were analyzed by ELISA and results are presented in Supplementary Information Fig. S3. Lung histology was performed to assess lung injury induced by inhalation exposures. Control mice exposed to filtered air exhibited no pulmonary histopathology after a single 3-h inhalation exposure (Fig. 4G). CB exposed mice also exhibited no lung histopathology except a significant increase in particle laden macrophages. In contrast animals exposed for 3 h to O_3_ had mild multifocal areas of bronchiolar epithelial cell necrosis with exfoliation near some airway branching sites in proximal large-diameter and small-diameter preterminal bronchioles throughout the lung lobes. Similar epithelial necrosis was evident in a few terminal bronchioles. Mice that received a single 3-h co-exposure to CB + O_3_ had multifocal areas of bronchiolar epithelial necrosis in preterminal and terminal bronchioles. In addition, they had a significant number of particle laden macrophages.

### 3.4 Co-exposure induces significantly greater respiratory function decline

**Fig. 5:**
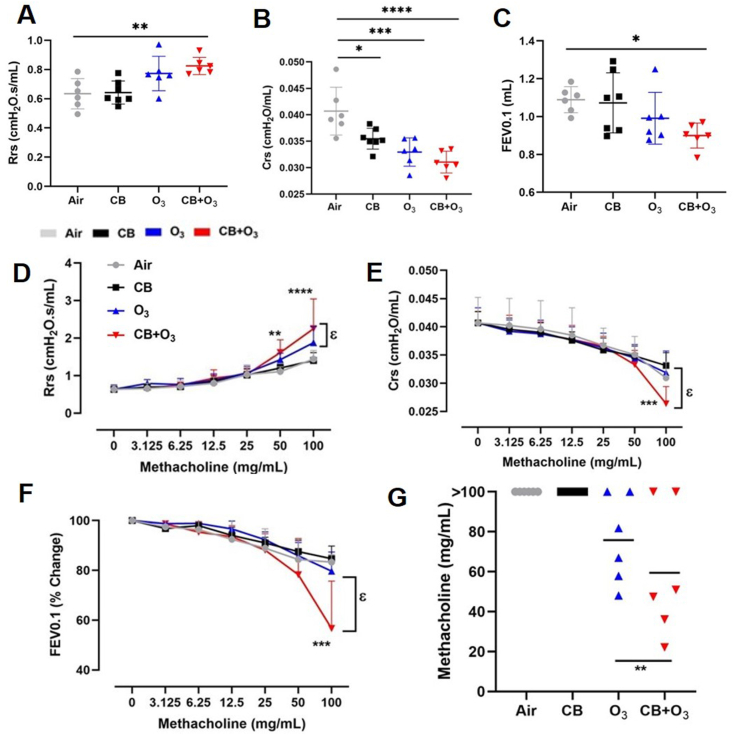
**Co-exposure induces significantly greater lung function decline.** Lung function assessment by FlexiVent. Baseline measurements (without methacholine provocation) of **A**) total respiratory resistance (Rrs), **B**) dynamic compliance (Crs) of the respiratory system and, **C**) forced expiratory volume at 0.1s (FEV0.1s) at 24 h post single (3 h) exposure to either air, CB (10 mg/m^3^), O_3_ (2 ppm) or CB + O_3_ (10 mg/m^3^ + 2 ppm). Methacholine dose response for forced oscillation technique (FOT) parameters **D**) total respiratory resistance (Rrs) and **E**) dynamic compliance (Crs) of the respiratory system. Methacholine dose response for forced expiration parameter F) forced expiratory volume at 0.1s (FEV0.1s). G) provocative concentration 20 (PC20) calculation for FEV0.1. Data are presented as mean ± SD of n = 8–10 mice per group and analyzed by two-way analysis of variance (ANOVA) followed by Tukey's post hoc test. *p < 0.05, *p < 0.01, ***p < 0.001. ϵ denotes different between exposures at the same dose of methacholine.

Respiratory function measurements using the Flexivent® system demonstrated a significant increase in resistance (Rrs) as well as a decrease in compliance (Crs) and forced expiratory flow at 0.1 s (FEV0.1) in co-exposure mice compared with individual exposure groups (Fig. 5 A-C). Fig. 5 A-C show baseline data collected 24 h post-exposure without methacholine provocation. A dose response of methacholine indicated increased hyperreactivity in co-exposure mice (Fig. 5 D-F). Methacholine dose responses data was baseline normalized to highlight differences in methacholine sensitivity. However, O_3_ and co-exposure induced similar increases in airway Newtonian resistance (Rn) after provocation indicating that large airway hyperresponsiveness is mainly mediated by O_3_ exposure (Supporting Information Fig. S4). Moreover, a significant decline in provocative concentration 20 (PC20) of methacholine was observed in co-exposure mice compared with individual exposure groups (Fig. 5 G).

### 3.5 Modulation of oxidant generation protects from Co-exposure induced lung damage

**Fig. 6:**
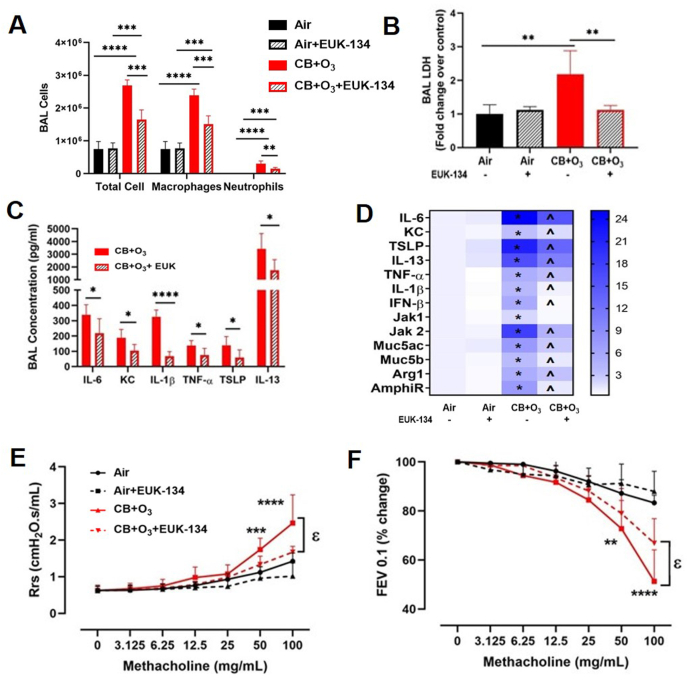
**Inhalation co-exposure induce oxidant dependent inflammation and lung function decline in mice.** Broncho-alveolar lavage analyses **A)** total cells, macrophages and neutrophils and **B**) LDH **C**) BAL fluid ELISA without or with 10 mg/kg EUK-134 pretreatment. **D**) lung tissue real-time PCR mRNA expression, and Lung function analyses **E**) total respiratory resistance **F**) FEV0.1. Data are presented as mean ± SD of n = 5–7 mice per group and analyzed by two-way analysis of variance (ANOVA) followed by Tukey's post hoc test. PCR values are presented as fold change values. * represents significantly different from control while ^ presents significantly different between EUK-134 in co-exposure group. *p < 0.05, *p < 0.01, ***p < 0.001. ϵ denotes different between exposures at the same dose of methacholine.

EUK-134, a synthetic superoxide dismutase catalase mimetic, was utilized in mice to elaborate the role of oxidant generation. Animals injected with EUK-134 had significantly lower lung inflammation (BALF total cells, macrophages, neutrophils) and lung cell death (LDH levels) (Fig. 6 A, B) compared to co-exposure alone. Moreover, co-exposure in the presence of EUK-134 lead to a less increase in protein and mRNA expression of markers related to inflammation and mucins as measured by ELISA and real time PCR (Fig. 6 C, D). One of the significantly altered oxidants mediated alarmin molecule was TSLP. Moreover, less increase in respiratory resistance and less decrease in FEV0.1 were observed after EUK-134 administration (Fig. 6 E, F). However, EUK treatment did not show benefit in terms of lavage protein contents (indictive of air-blood barrier integrity) (Supplementary Fig. S5). As EUK-134 was dissolved in water, we ran vehicle control groups for air and CB + O_3_ and the complete data set including these groups can be found in Supplementary Figs. S5 and S6. For clarity of presentation, we have not shown these in the original manuscript but have shown them in supplementary data.

### 3.6 TSLP pathway contributes towards lung inflammation induction by Co-exposure

**Fig. 7:**
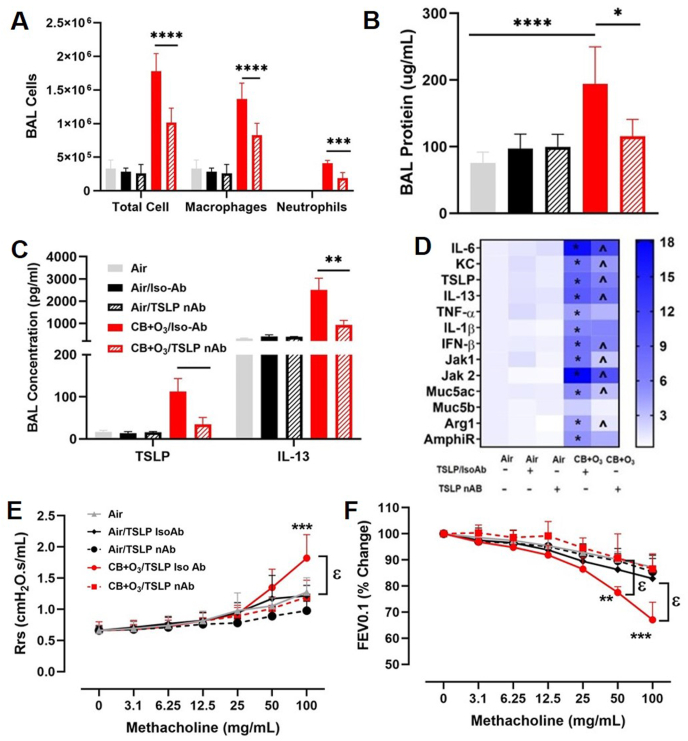
**Neutralization of TSLP significantly reduces the inflammatory effects of CB + O**_**3**_**inhalation co-exposures.** Mice were administered 25 μg of TSLP neutralizing antibody by pulmonary routes, inhalation exposures done for 3 h and scarified 24 h post exposure. Broncho-alveolar lavage analyses E**)** total cells, macrophages and neutrophils, **B**) total proteins, and **C**) ELISA. **D**) lung tissue real-time PCR mRNA expression. Lung function measurements **A)** Rrs **F**) FEV0.1 Data are presented as mean ± SD of n = 5–7 mice per group and analyzed by two-way analysis of variance (ANOVA) followed by Tukey's post hoc test. PCR values are presented as fold change values. * represents significantly different from air-isotype control while ^ presents significantly different between isotype and neutralizing antibody in co-exposure group. *p < 0.05, *p < 0.01, ***p < 0.001, ****p < 0.0001. ϵ denotes different between exposures at the same dose of methacholine.

TSLP neutralizing antibody was utilized to elucidate the role of TSLP production in IL-13 signaling pathway induction. Mice instilled with a TSLP neutralizing antibody demonstrated a significantly weaker inflammatory response after co-exposure (BALF total cells, macrophages and neutrophils) (Fig. 7 A). TSLP neutralization also had a protective effect on lung permeability, measured as BALF proteins, induced by co-exposure (Fig. 7 B). The effectiveness of TSLP neutralization was verified by ELISA assay (Fig. 7 C). TSLP neutralization led to a less increase in the levels of IL-13 protein after co-exposure (Fig. 7 C) and a significantly attenuated mRNA expression of a number of signaling molecules involved in inflammatory pathways (IL-6, KC, IL-13, JAK1/2, IFN-β), M2 macrophage marker (arginase), and mucins (Muc5ac) (Fig. 7 D). Moreover, TSLP neutralization resulted in a significantly less changes in respiratory function parameters (Rrs and FEV0.1) (Fig. 7 E, F). Mechanistically, oxidant-free radical production and oxidant-induced cytokine (IL-1β, TNF-α) production are an upstream event and was not anticipated to change after TSLP neutralization. TSLP neutralization however did not alter the cytotoxicity and some inflammatory cytokines further indicating specific IL-13 pathway modulation (Supplementary Fig. S7).

## 4 Discussion

This study validates a novel inhalation co-exposure system and establishes the increased reactivity/toxicity of ultrafine particle and O_3_ co-exposure using a physiological relevant inhalation exposure route. Utilization of engineered ultrafine CB as a representative of the carbon core of pollution particles allowed to rule out the varying contribution of contaminating organic and inorganic components and allowed for the creation of a model system in which further contaminants could be added to the study their specific impacts. We intentionally chose CB over black carbon (BC), a constituent of environmental pollution which contains high amounts of adsorbed environmental contaminants (polycyclic aromatic hydrocarbons, lipopolysaccharide, metals and allergens), because the latter has highly variable composition, making it impossible to determine the contribution of individual components [45]. In addition, CB is among the top five highly produced engineered nanomaterials for consumer product applications [12,46]. These applications, which include printer toners, rubber tires, paints etc., also have significant potential of adding inhalable ultrafine carbon into the environment.

Inhalation co-exposure to CB and O_3_ induced a greater than additive effect as calculated by the following basic criteria of interaction [(CB-sham) + (O_3_-sham) < (CB + O_3_ co-exposure - sham)]. Our data confirm these interactions in terms of multiple parameters related to lung injury, lung function, and mRNA/protein expression of cytokines. Due to the limitation that CB alone does not induce significantly different response from control, it is impossible to distinguish between synergistic and permissive effects with this single dose study. This limits the ability to apply a more stringent statistical approach for synergy estimation. The question of synergy is currently being explored using the in vitro systems as doing extensive dose response studies with animal models is not feasible.

Previous studies reported additive or more than additive, pulmonary toxic impacts of O_3_ and particulate matter/diesel exhaust particle (DEP) co- and/or sequential exposure in human volunteers and animal models [13,26,[47], [48], [49], [50]]. These studies provided valuable information indicating interactions between O_3_ and particulates. However, PM/DEP itself is a heterogenous mixture of air pollution components such as elemental and organic carbon, hydrocarbons, metals, endotoxin, and allergens. Moreover, interaction with O_3_ can lead to chemical modifications of particle surfaces rendering them more biologically active [13,26,51]. Taken together, this clearly laid the foundation for studies to identify the contribution of individual components of PM/DEP in co-exposure toxicity. Our study not only recapitulates the additive/more than additive pulmonary toxicity observed from O_3_ and PM/DEP co-exposure but also further clarifies the contribution of the particulate core/elemental carbon. Moreover, the study provided the foundations for an experimental system in which further constituents can be added and evaluated in a controlled fashion.

Acellular oxidant generation is a consistent feature of airborne particulate matter [[52], [53], [54]]. Recently, lung antioxidant depletion was proposed as a strong indicator of ambient fine particulate matter-induced cellular stress [55]. We and others have previously demonstrated that ultrafine/nano CB particles exhibit the ability to generate oxidants under acellular conditions [37,56]. Moreover, O_3_-interacted CB particles were reported to demonstrate acellular oxidant generation potentials [29,51]. In a recent report demonstrating increased inflammatory reactivity of O_3_-interacted CB particles after intratracheal instillation, fluidized bed approach was used to generate O_3_-interacted particles which were used for intratracheal instillation to elaborate increased inflammatory potential [29]. A recent study described that O_3_ alters the composition of the PM and increases its potency to induce lung injury in spontaneously hypertensive rats [26]. Herein, our EPR and FRAS data validate these interactions at significantly lower concentrations of pollutants and subsequent oxidant/free radical generation on aerosolized particles. Our EPR spectroscopic studies of solid samples in solution demonstrate that the active surfaces on the CB are available for reaction. EPR spin trapping experiments have shown that CB particles do not generate superoxide and hydroxyl radical in PBS. In addition to providing mechanistic understanding of respiratory function decline following acute air pollution episode (requiring higher exposure doses), our data confirm interactive oxidant generation at levels consistently observed in highly polluted cities and levels routinely used in human clinical/volunteer studies (250 μg/m^3^ CB and 200 ppb O_3_) to assess air pollution effects.

We validate the biological relevance of acellular oxidant generation in induction of pulmonary damage (inflammation, and function decline) by demonstrating a significant mitigation of adverse effects after administration of EUK-134. Treatment with EUK-134 previously demonstrated benefits in a variety of oxidant-induced pathologic processes such as myocardial ischemia reperfusion injury, pulmonary hypertension, chronic kidney disease and ischemic brain injury [[57], [58], [59], [60]]. However, its impact on lung inflammation, airway hyperreactivity (AHR) and overall respiratory function stemming from oxidant generation after inhalation of air pollution components was not known earlier. Here, we demonstrate benefits of EUK-134 administration after inhalation co-exposure to CB and O_3_ in terms of multiple pulmonary endpoints including BAL cellularity, lung injury and functional indices. A significant lower increase in inflammatory mediator(s) mRNA and protein expression further confirms the involvement of ROS in CB + O_3_ co-exposure induced lung damage.

Epithelial alarmin (TSLP)-induced IL-13 pathway was observed to be among the significantly perturbed pathways that was ameliorated by EUK-134 administration. TSLP is mainly produced by epithelial cells in allergic disorders such as asthma and atopic dermatitis [7,8,[61], [62], [63]]. Association between TSLP and disease severity has been reported in asthmatics [64]. One of the effector pathways through which TSLP exerts its roles is by regulating IL-13 expression [61,65]. IL-13 is critical in regulating inflammatory and immune responses [66]. IL-13 creates a typical Th2 milieu in the lungs which favors the propagation of chronic inflammatory disorders and mediates exhibition of characteristic allergic hallmarks such eosinophilia, mucous cell metaplasia, immunoglobulin E production, airway hyperresponsiveness and fibrosis [67]. In addition, IL-13 is also an alternate macrophage (M2) phenotype inducer [68,69]. Importantly, our data demonstrating an increased arginase-1 expression in the lung confirms this outcome. Moreover, IL-13Rα1 is principal mediator of IL-13 induced changes mentioned above in experimental asthma [70]. Furthermore, IL-13 has proven roles in mucous cell metaplasia and our results clearly indicate increase expression of mucous production genes (Muc5b and Muc5ac) after co-exposures, which are significantly reduced by TSLP neutralization (which leads to significant reductions in IL-13 expression). It is well known that in chronic obstructive lung disorders increase mucous production and lead to impaired lung function and air flow limitation, a characteristic feature observed in our lung function studies.

The Occupational Safety and Health Administration (OSHA) airborne permissible exposure limit (PEL) for CB is 3.5 mg/m^3^ averaged over 8-h work shift. However, workplace exposure levels of 79 mg/m^3^ to 675 mg/m^3^ have been reported [11,71,72]. Our single exposure (3 h) lead to a measured pulmonary deposited dose of 2.2 μg. Multiple Path Particle Dosimetry Model (MPPD v3.04) predicted 12.4% human pulmonary deposition fraction for CB exposure [73]. Therefore, using average worker parameters, a daily deposited dose of 4.17 mg was estimated:

Factored for human dose using OSHA PEL of 3.5 mg/m^3^:

Aerosol concentration x min volume x exposure duration deposition efficiency = deposited human dose.

3.5 mg/m^3^ x (20L/min) (10^−3^ m^3^/L) x (8 h/day) x 60 min/h x 0.124 = 4.17 mg deposited/8 h in a worker.

Human equivalent to mouse measured deposited dose by surface area (SA):

(SA _human_ x Lung Burden _mouse_)/ SA _mouse_ = Lung Burden _human_

(102 m^2^ × 0.0022 mg)/0.05 m^2^ = 4.5 mg Therefore, our daily deposited dose in mouse will not be very different than what could be estimated from a worker in conditions of 3.5 mg/m^3^ for 8 h. Deposited dose in mouse corresponds to 35 days exposure of 35 μg/m^3^ national ambient air quality standard (NAAQS) for PM2.5 for 24 h. Given that urban global PM_2.5_ average levels routinely exceed 35 μg/m^3^ and approximately 90% of the urban population is exposed to concentrations exceeding the World Health Organization air quality guidelines, the exposure levels in this study are certainly relevant. The O_3_ (2 ppm for 3 h) was based on a similar biological response outcome in exercising human and previously published deposition in rodent models [[74], [75], [76], [77], [78]]. It is important to note that because of the differences in anatomy of the respiratory tract and sedentary nature of laboratory rodents, a significantly higher (4–5 times) exposure dose translates to the comparable effects induced in exercising human subjects under controlled acute exposure conditions [75,79]. Taken this into consideration, our O_3_ dose translates to an approximately 4–5 times the effective dose (400 ppb) utilized in controlled exposure human studies that leads to pulmonary neutrophilia [75]. Given that standards are based on timed averages, and they are often exceeded, we find our exposure levels/model highly relevant to human exposures.

An accelerated lung function decline is observed in subjects living in highly polluted areas compared with less polluted areas [80]. Classically, lung function is assessed using forced oscillation technique (FOT) that measures resistance and compliance and can distinguish between the airway and tissue mechanics [34,38,81]. These measurements have been used extensively in animal models of pulmonary disease to characterize lung mechanical responses [34,38,81,82]. A recently developed negative pressure-driven forced expiration (NPFE) technique measures forced expiration (FE) parameters in a manner similar to human spirometry [38]. We previously demonstrated nanoparticles of titanium dioxide and gold can significantly increase AHR in a mouse model of asthma [34] and recently reported lung function changes in multiple pulmonary disease models including after O_3_ exposure [39,83]. Herein, we demonstrate that CB + O_3_ co-exposure induced significant AHR and pulmonary function decline both in terms of FOT and FE parameters. Interestingly, we observed similar significant increases in airway Newtonian resistance (Rn) in the case of CB + O_3_ co-exposure and O_3_ indicating that the large airway resistance response was mainly caused by O_3_. In contrast, total respiratory resistance was increased more after co-exposure than individual CB or O_3_ exposures. FE parameter FEV0.1 (even without methacholine provocation) demonstrated an aggravated obstructive phenotype after co-exposure. Indeed, TSLP gene variants have been associated with lower lung function in healthy individuals as well as a susceptibility locus to asthma [[84], [85], [86]]. An association between plasma concentration of IL-13 and increasing severity of airflow obstruction and diffusion capacity of carbon monoxide (DLCO) was reported in human subjects suffering from chronic obstructive pulmonary disease [87]. Moreover, IL-13 has well known roles in airway mucin production, mucous cell metaplasia and inflammation in both humans as well as animal models of allergic disorders [[88], [89], [90], [91], [92]].

**Fig. 8:**
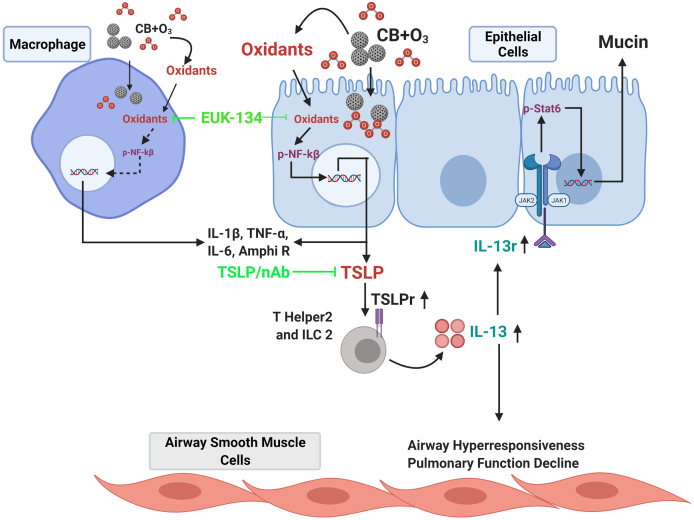
Overview figure representing a pathway for co-exposure induced pulmonary inflammation and lung function decline.

In summary, this study provided evidence that inhalation co-exposures closely replicate air pollution-induced alterations in lung inflammation and lung function decline. Here, by providing initial proof of this concept, we propose revisiting single pollutant mass-based regulatory guidelines for air pollution. Moreover, results from this study delineates changes in a key mechanistic pathway (oxidant-TSLP-IL-13 axis) by inhalation of air pollution components and quantifies increased lung inflammation and lung function decline potentials of particle and gas inhalation co-exposures after an acute inhalation exposure. A schematic of this mechanistic pathways is presented in Fig. 8. Further research utilizing chronic exposure scenarios, susceptibility models, and adding more pollutants into the co-exposure to study the complex interactions between components of air pollution, is warranted.

## Disclaimer

The findings and conclusions in this report are those of the author(s) and do not necessarily represent the official position of the National Institutes of Health and National Institute for Occupational Safety and Health, Centers for Disease Control and Prevention. Mention of brand name does not constitute product endorsement.

## Funding

This study was supported by National Institute of Health funding R01 ES031253 (SH), NIGMS U54GM104942 (SH), R01 ES015022 (TRN), R01 DK124510 (EEK), P20 GM109098 (EEK), NIA R56 NS117754 (EEK), and NIOSH NTRC # 9390BN6 (VK).

## Note

Authors declare no competing financial interest.

## CRediT authorship contribution statement

**Nairrita Majumder:** Methodology, Investigation, Formal analysis, Visualization, Writing – original draft. **William T. Goldsmith:** Methodology, Investigation, Formal analysis, Software, Visualization, Writing – original draft. **Vamsi K. Kodali:** Methodology, Investigation, Formal analysis, Visualization, Writing – original draft. **Murugesan Velayutham:** Investigation, Formal analysis, Visualization, Writing – original draft. **Sherri A. Friend:** Investigation, Formal analysis. **Valery V. Khramtsov:** Methodology, Writing – original draft. **Timothy R. Nurkiewicz:** Methodology, Writing – original draft. **Aaron Erdely:** Methodology, Writing – original draft. **Patti C. Zeidler-Erdely:** Methodology, Writing – original draft, Methodology. **Vince Castranova:** Conceptualization, Writing – original draft. **Jack R. Harkema:** Investigation, Formal analysis, Writing – original draft. **Eric E. Kelley:** Methodology, Investigation, Writing – original draft. **Salik Hussain:** Conceptualization, Methodology, Investigation, Formal analysis, Visualization, Writing – original draft, Supervision, Project administration, Funding acquisition, All authors read and approved the final manuscript.

## Declaration of competing interest

The authors declare that they have no known competing financial interests or personal relationships that could have appeared to influence the work reported in this paper.
