# Supplementary material for: Oxidant-induced epithelial alarmin pathway mediates lung inflammation and functional decline following ultrafine carbon and ozone inhalation co-exposure
Source: Redox Biol. 2021 Aug 5;46:102092. doi: 10.1016/j.redox.2021.102092 (PMC8385153; doi:10.1016/j.redox.2021.102092)
Supplement: Multimedia component 1 [file mmc1.pptx]

## Slide 1
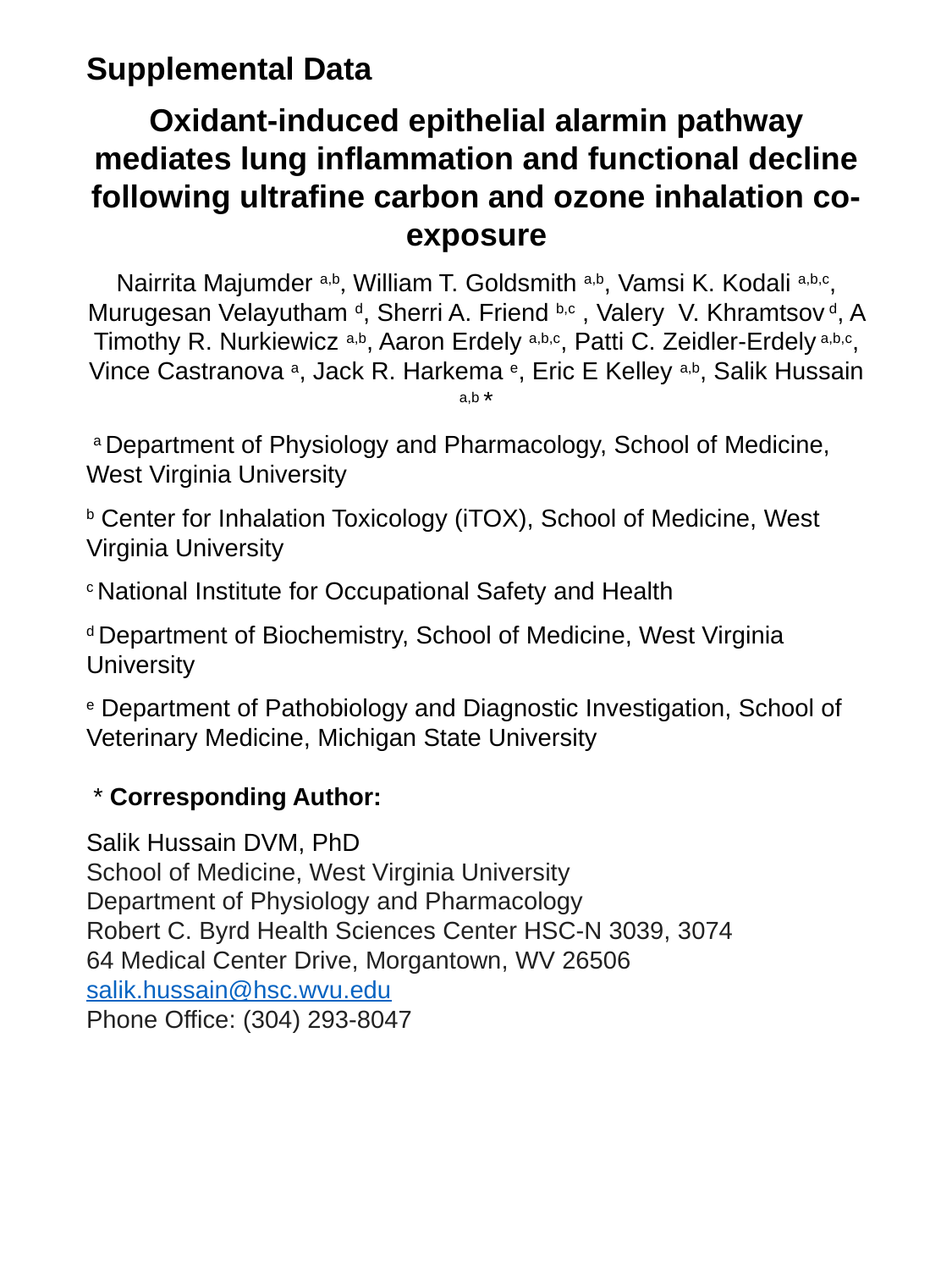

Supplemental Data
Oxidant-induced epithelial alarmin pathway mediates lung inflammation and functional decline following ultrafine carbon and ozone inhalation co-exposure
Nairrita Majumder a,b, William T. Goldsmith a,b, Vamsi K. Kodali a,b,c, Murugesan Velayutham d, Sherri A. Friend b,c , Valery V. Khramtsov d, A Timothy R. Nurkiewicz a,b, Aaron Erdely a,b,c, Patti C. Zeidler-Erdely a,b,c, Vince Castranova a, Jack R. Harkema e, Eric E Kelley a,b, Salik Hussain a,b *
 a Department of Physiology and Pharmacology, School of Medicine, West Virginia University
b Center for Inhalation Toxicology (iTOX), School of Medicine, West Virginia University
c National Institute for Occupational Safety and Health
d Department of Biochemistry, School of Medicine, West Virginia University
e Department of Pathobiology and Diagnostic Investigation, School of Veterinary Medicine, Michigan State University
 * Corresponding Author:
Salik Hussain DVM, PhD
School of Medicine, West Virginia University
Department of Physiology and Pharmacology
Robert C. Byrd Health Sciences Center HSC-N 3039, 3074
64 Medical Center Drive, Morgantown, WV 26506
salik.hussain@hsc.wvu.edu
Phone Office: (304) 293-8047

## Slide 2
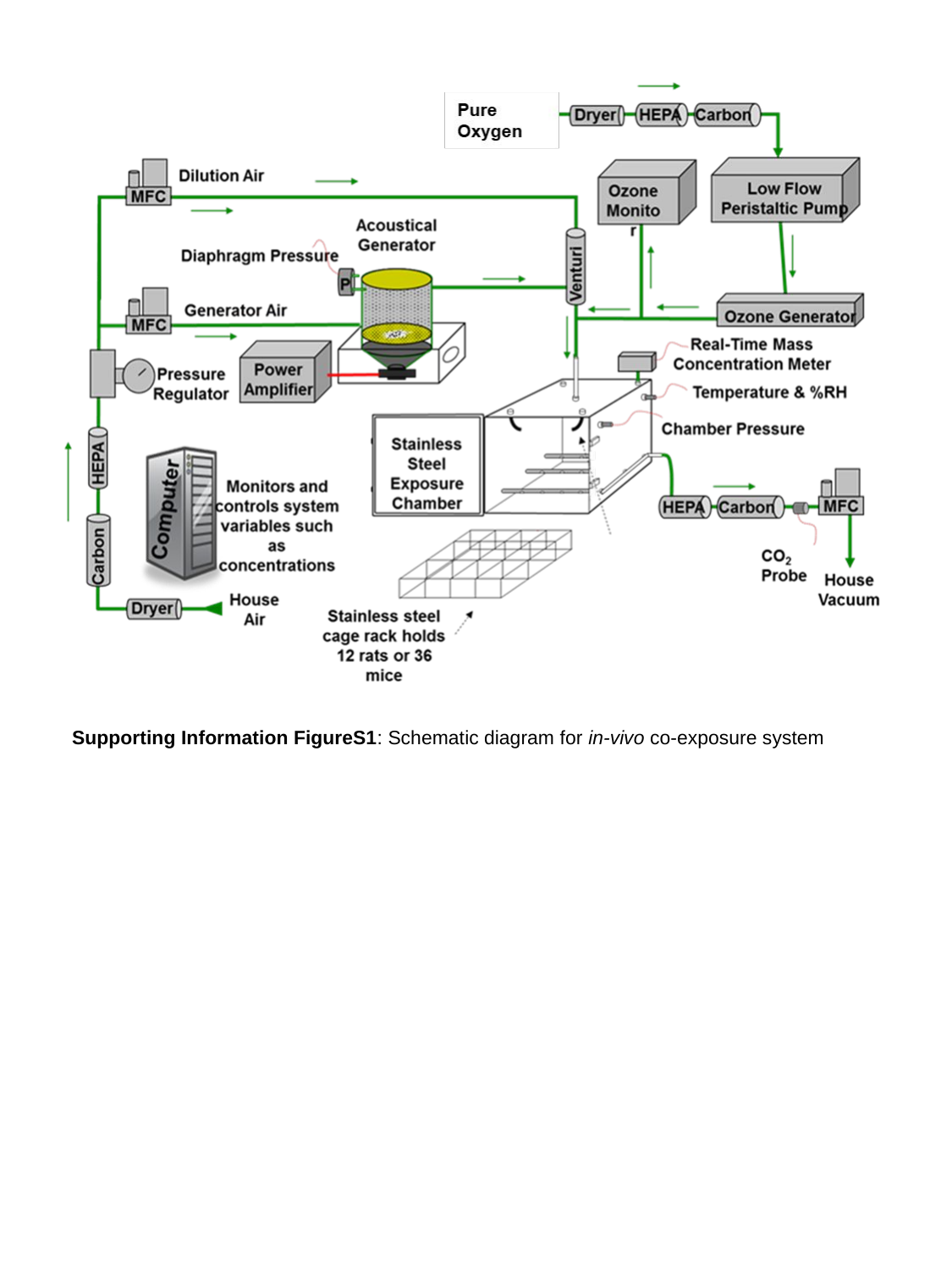

Supporting Information FigureS1: Schematic diagram for in-vivo co-exposure system

## Slide 3
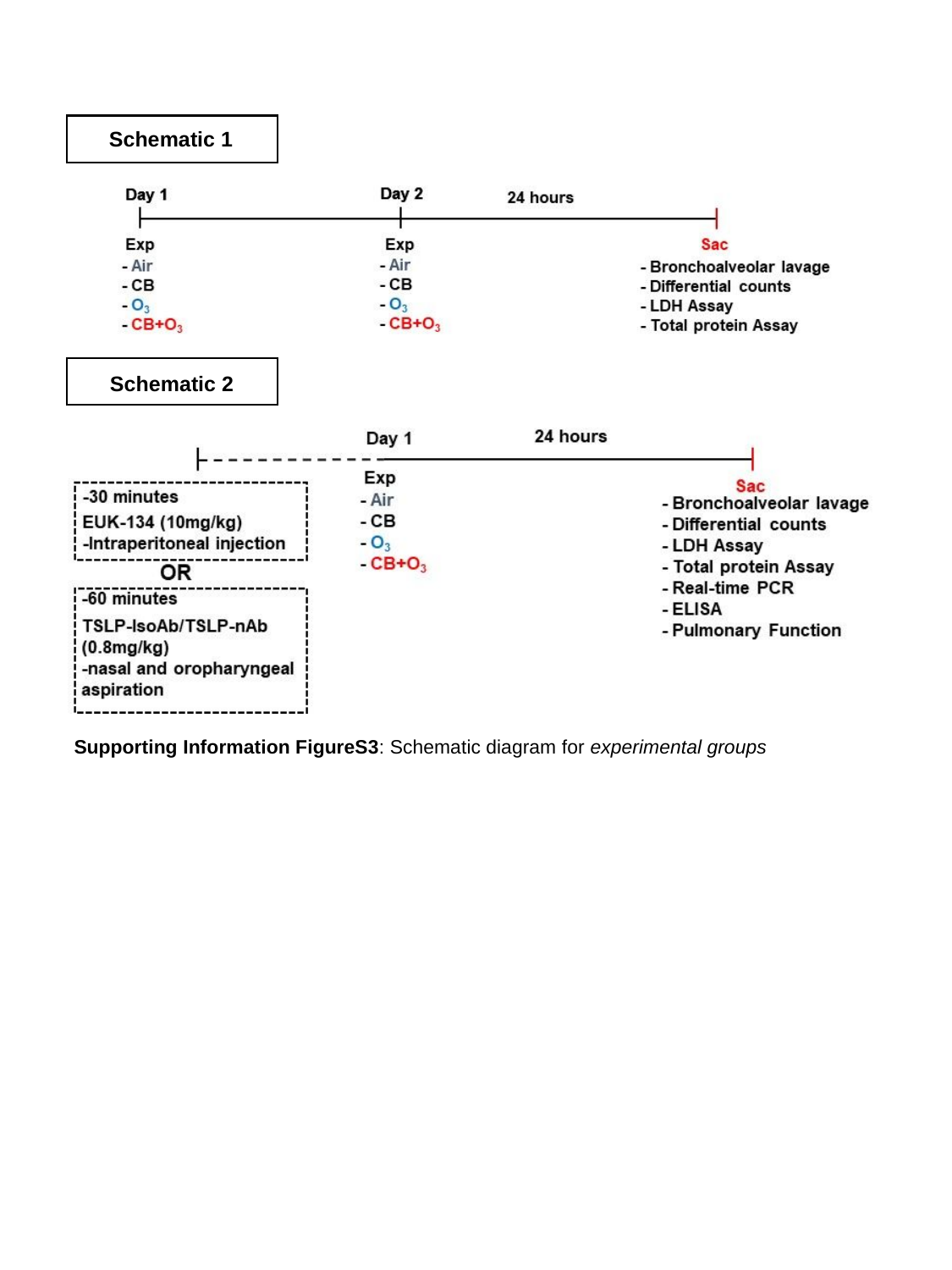

Schematic 1
Schematic 2
Supporting Information FigureS3: Schematic diagram for experimental groups

## Slide 4
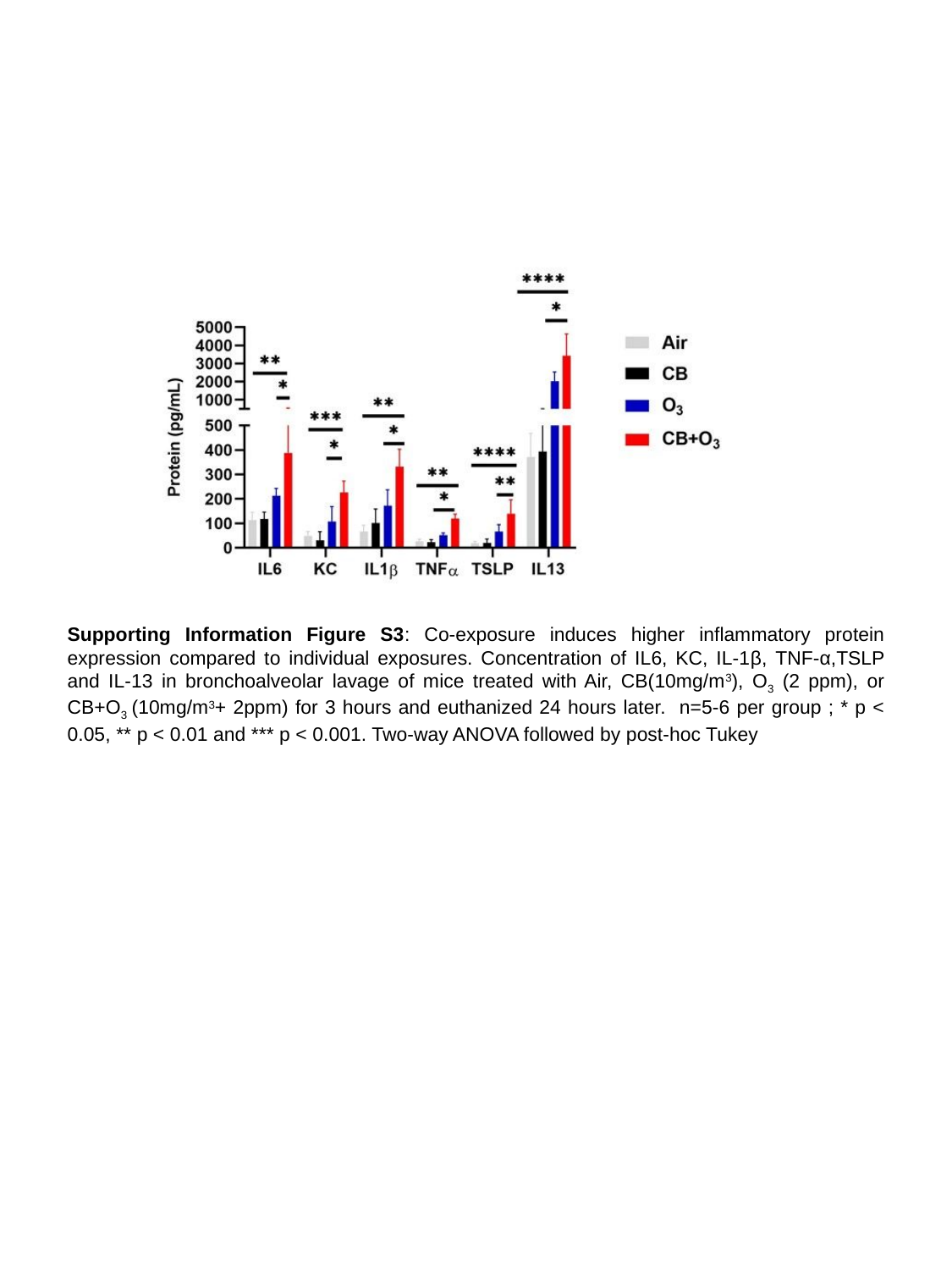

Supporting Information Figure S3: Co-exposure induces higher inflammatory protein expression compared to individual exposures. Concentration of IL6, KC, IL-1β, TNF-α,TSLP and IL-13 in bronchoalveolar lavage of mice treated with Air, CB(10mg/m3), O3 (2 ppm), or CB+O3 (10mg/m3+ 2ppm) for 3 hours and euthanized 24 hours later. n=5-6 per group ; * p < 0.05, ** p < 0.01 and *** p < 0.001. Two-way ANOVA followed by post-hoc Tukey

## Slide 5
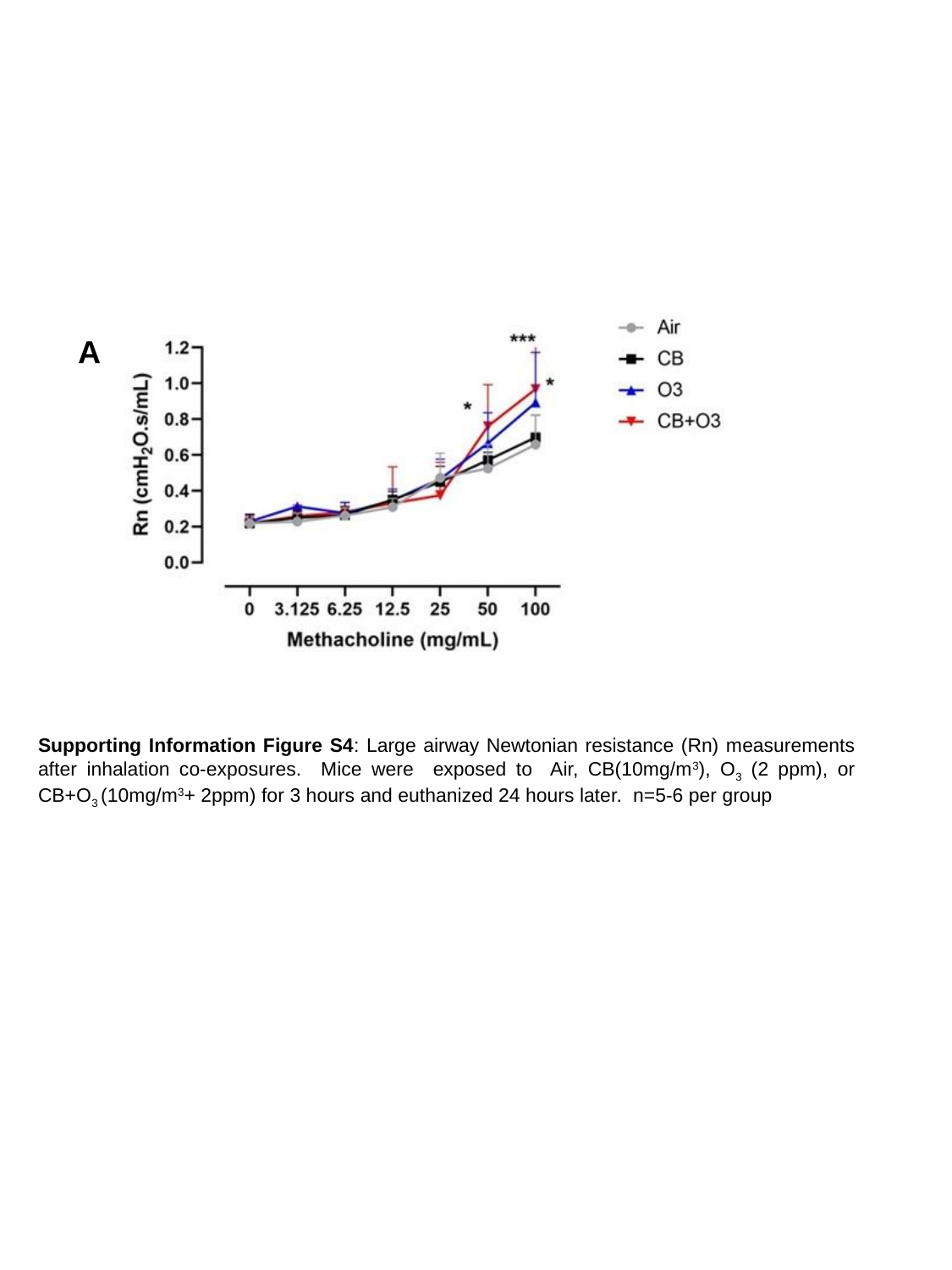

A
Supporting Information Figure S4: Large airway Newtonian resistance (Rn) measurements after inhalation co-exposures. Mice were exposed to Air, CB(10mg/m3), O3 (2 ppm), or CB+O3 (10mg/m3+ 2ppm) for 3 hours and euthanized 24 hours later. n=5-6 per group

## Slide 6
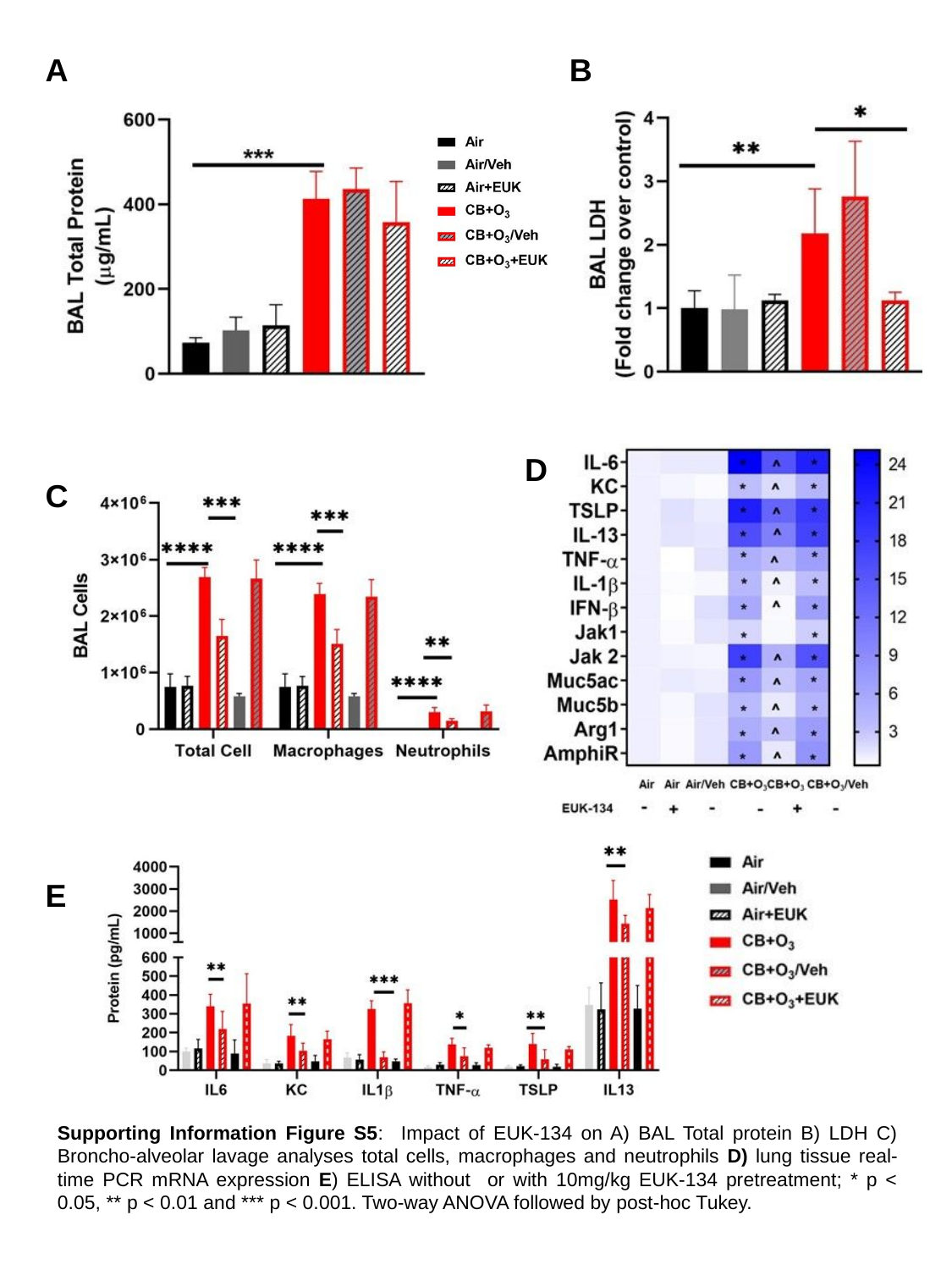

A
B
D
C
E
Supporting Information Figure S5: Impact of EUK-134 on A) BAL Total protein B) LDH C) Broncho-alveolar lavage analyses total cells, macrophages and neutrophils D) lung tissue real-time PCR mRNA expression E) ELISA without or with 10mg/kg EUK-134 pretreatment; * p < 0.05, ** p < 0.01 and *** p < 0.001. Two-way ANOVA followed by post-hoc Tukey.

## Slide 7
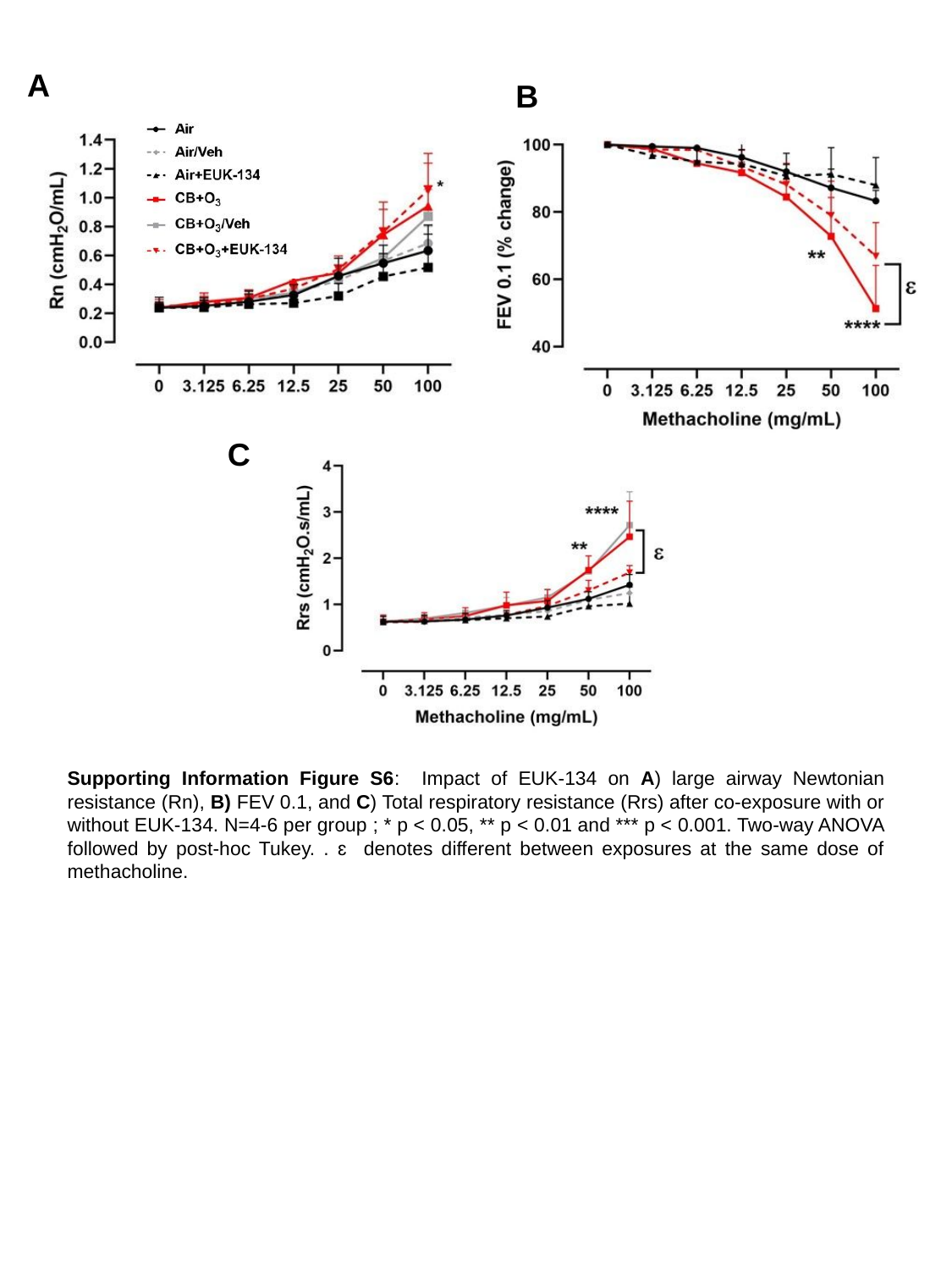

A
B
C
Supporting Information Figure S6: Impact of EUK-134 on A) large airway Newtonian resistance (Rn), B) FEV 0.1, and C) Total respiratory resistance (Rrs) after co-exposure with or without EUK-134. N=4-6 per group ; * p < 0.05, ** p < 0.01 and *** p < 0.001. Two-way ANOVA followed by post-hoc Tukey. . ɛ denotes different between exposures at the same dose of methacholine.

## Slide 8
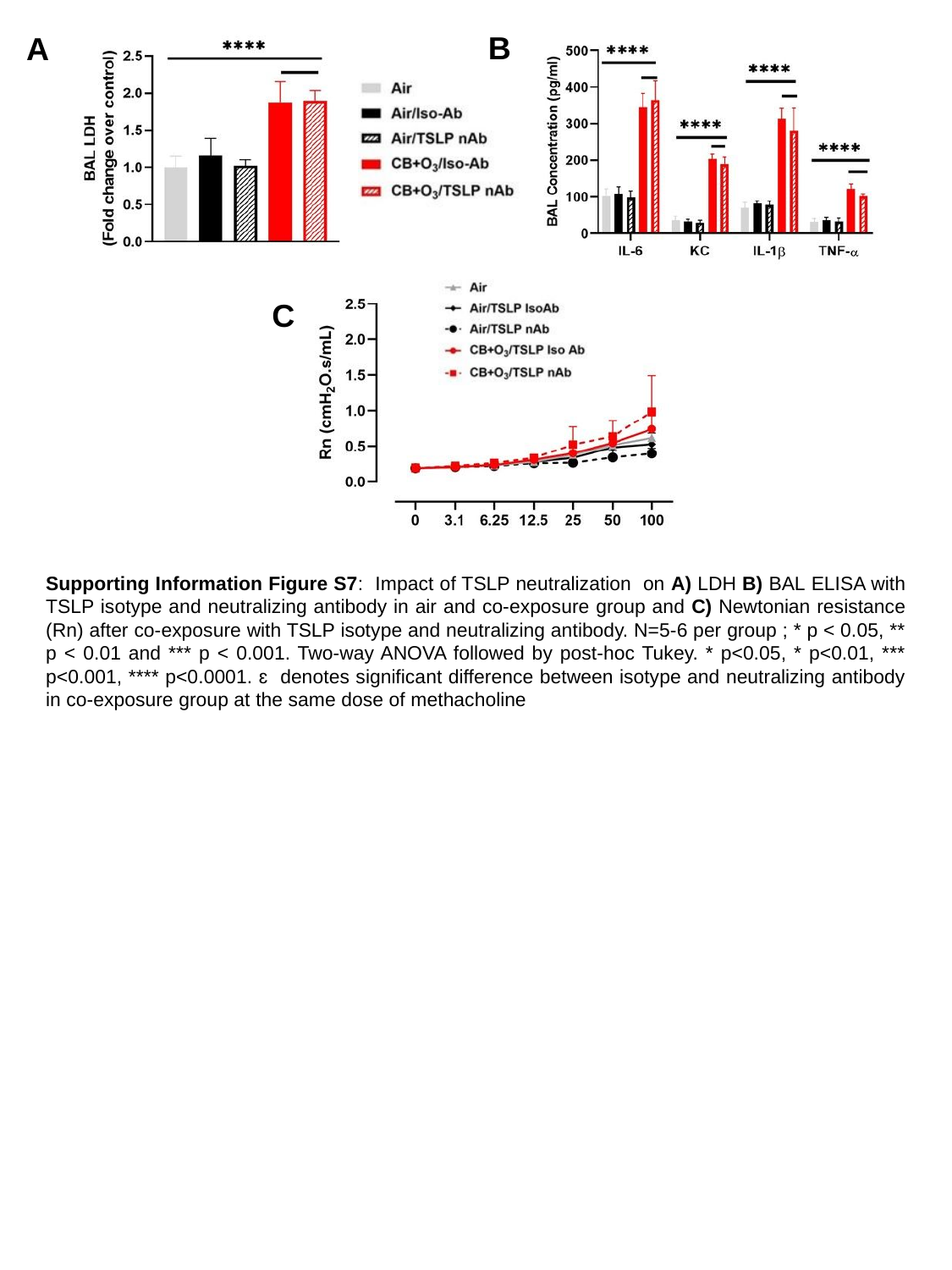

B
A
C
Supporting Information Figure S7: Impact of TSLP neutralization on A) LDH B) BAL ELISA with TSLP isotype and neutralizing antibody in air and co-exposure group and C) Newtonian resistance (Rn) after co-exposure with TSLP isotype and neutralizing antibody. N=5-6 per group ; * p < 0.05, ** p < 0.01 and *** p < 0.001. Two-way ANOVA followed by post-hoc Tukey. * p<0.05, * p<0.01, *** p<0.001, **** p<0.0001. ɛ denotes significant difference between isotype and neutralizing antibody in co-exposure group at the same dose of methacholine

## Slide 9
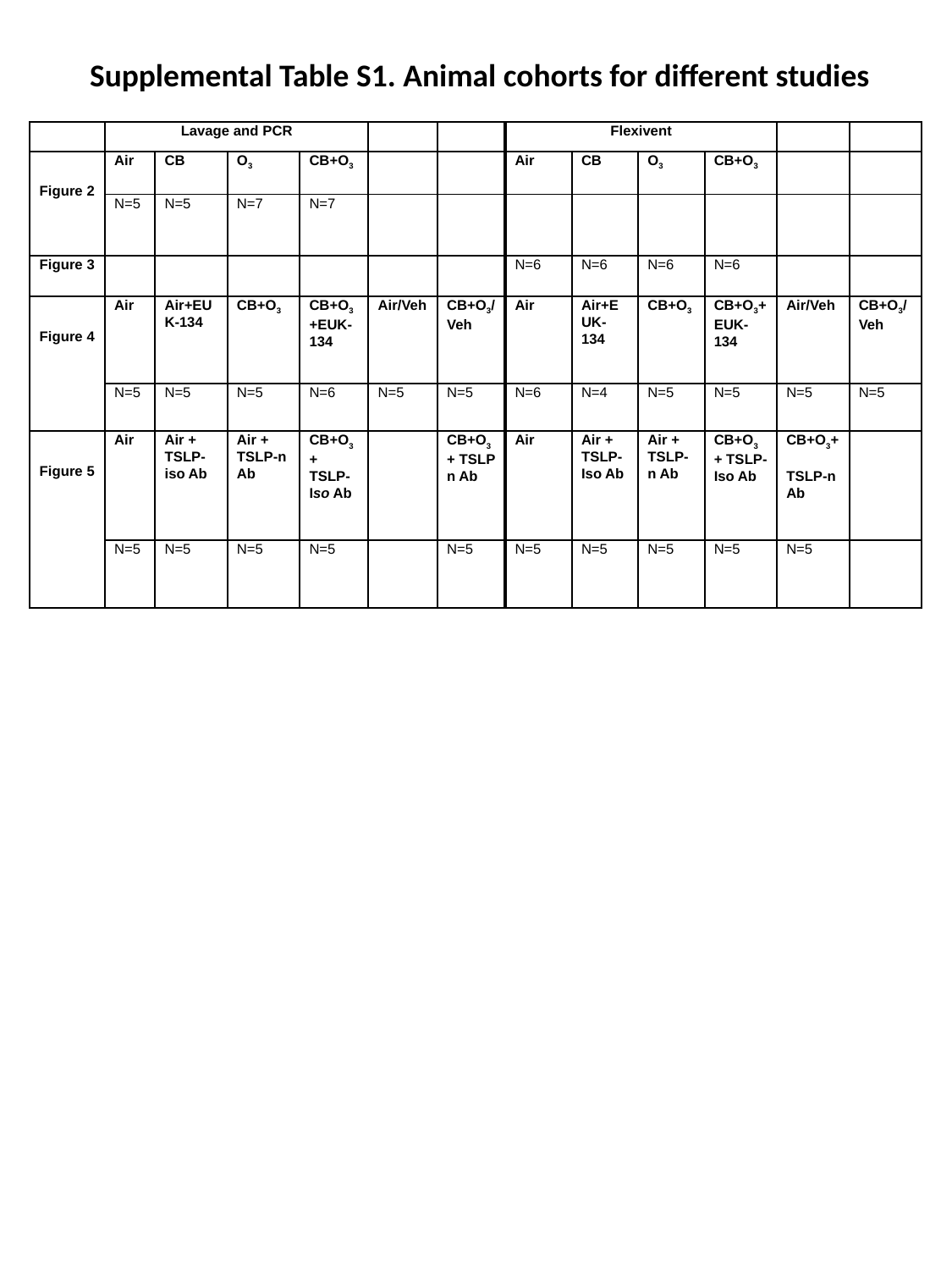

Supplemental Table S1. Animal cohorts for different studies
| | Lavage and PCR | | | | | | Flexivent | | | | | |
| --- | --- | --- | --- | --- | --- | --- | --- | --- | --- | --- | --- | --- |
| Figure 2 | Air | CB | O3 | CB+O3 | | | Air | CB | O3 | CB+O3 | | |
| | N=5 | N=5 | N=7 | N=7 | | | | | | | | |
| Figure 3 | | | | | | | N=6 | N=6 | N=6 | N=6 | | |
| Figure 4 | Air | Air+EUK-134 | CB+O3 | CB+O3+EUK-134 | Air/Veh | CB+O3/Veh | Air | Air+EUK-134 | CB+O3 | CB+O3+EUK-134 | Air/Veh | CB+O3/Veh |
| | N=5 | N=5 | N=5 | N=6 | N=5 | N=5 | N=6 | N=4 | N=5 | N=5 | N=5 | N=5 |
| Figure 5 | Air | Air + TSLP-iso Ab | Air + TSLP-n Ab | CB+O3 + TSLP-Iso Ab | | CB+O3+ TSLP n Ab | Air | Air + TSLP-Iso Ab | Air + TSLP-n Ab | CB+O3 + TSLP-Iso Ab | CB+O3+ TSLP-n Ab | |
| | N=5 | N=5 | N=5 | N=5 | | N=5 | N=5 | N=5 | N=5 | N=5 | N=5 | |

## Slide 10
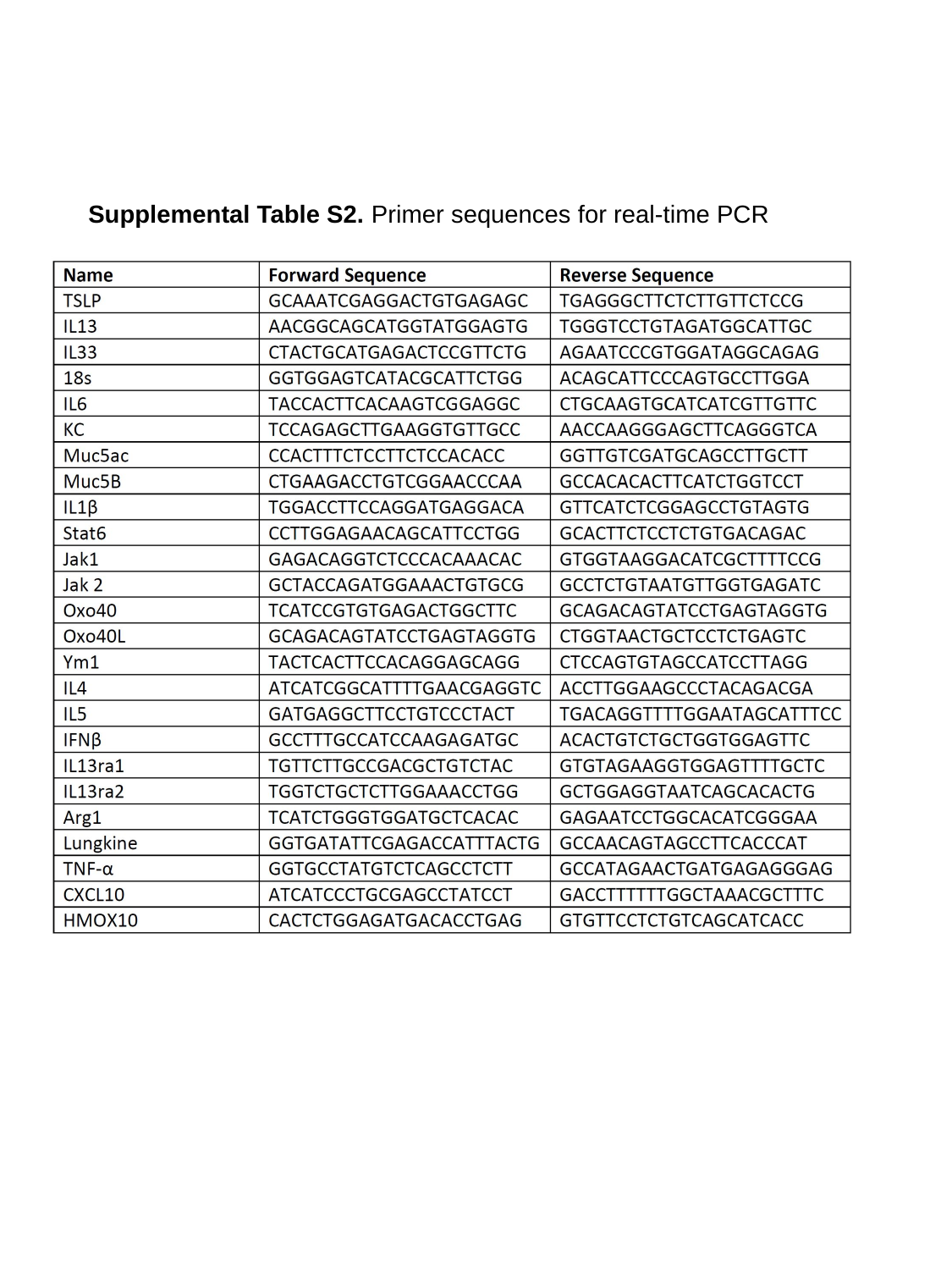

Supplemental Table S2. Primer sequences for real-time PCR

## Slide 11
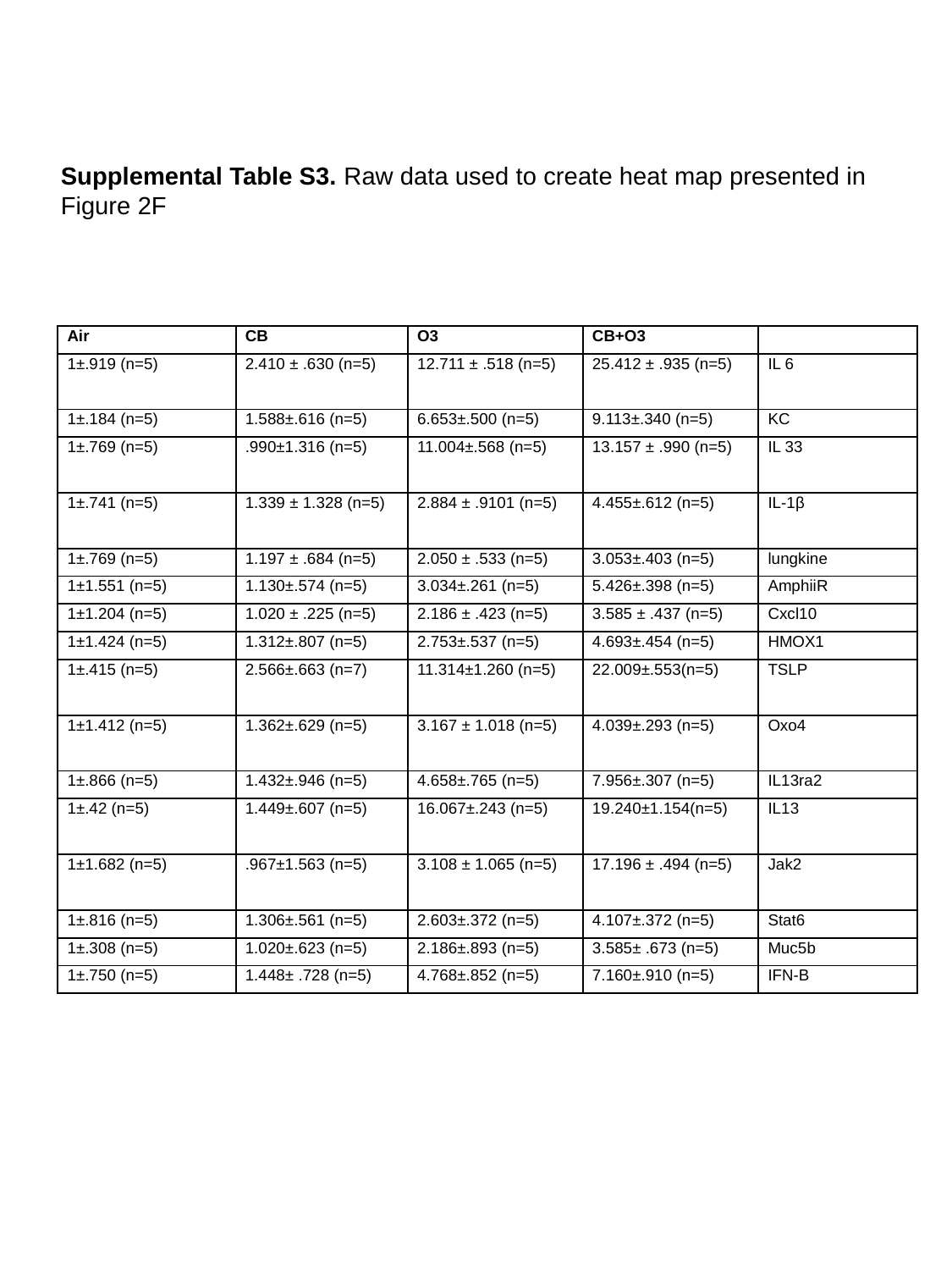

Supplemental Table S3. Raw data used to create heat map presented in Figure 2F
| Air | CB | O3 | CB+O3 | |
| --- | --- | --- | --- | --- |
| 1±.919 (n=5) | 2.410 ± .630 (n=5) | 12.711 ± .518 (n=5) | 25.412 ± .935 (n=5) | IL 6 |
| 1±.184 (n=5) | 1.588±.616 (n=5) | 6.653±.500 (n=5) | 9.113±.340 (n=5) | KC |
| 1±.769 (n=5) | .990±1.316 (n=5) | 11.004±.568 (n=5) | 13.157 ± .990 (n=5) | IL 33 |
| 1±.741 (n=5) | 1.339 ± 1.328 (n=5) | 2.884 ± .9101 (n=5) | 4.455±.612 (n=5) | IL-1β |
| 1±.769 (n=5) | 1.197 ± .684 (n=5) | 2.050 ± .533 (n=5) | 3.053±.403 (n=5) | lungkine |
| 1±1.551 (n=5) | 1.130±.574 (n=5) | 3.034±.261 (n=5) | 5.426±.398 (n=5) | AmphiiR |
| 1±1.204 (n=5) | 1.020 ± .225 (n=5) | 2.186 ± .423 (n=5) | 3.585 ± .437 (n=5) | Cxcl10 |
| 1±1.424 (n=5) | 1.312±.807 (n=5) | 2.753±.537 (n=5) | 4.693±.454 (n=5) | HMOX1 |
| 1±.415 (n=5) | 2.566±.663 (n=7) | 11.314±1.260 (n=5) | 22.009±.553(n=5) | TSLP |
| 1±1.412 (n=5) | 1.362±.629 (n=5) | 3.167 ± 1.018 (n=5) | 4.039±.293 (n=5) | Oxo4 |
| 1±.866 (n=5) | 1.432±.946 (n=5) | 4.658±.765 (n=5) | 7.956±.307 (n=5) | IL13ra2 |
| 1±.42 (n=5) | 1.449±.607 (n=5) | 16.067±.243 (n=5) | 19.240±1.154(n=5) | IL13 |
| 1±1.682 (n=5) | .967±1.563 (n=5) | 3.108 ± 1.065 (n=5) | 17.196 ± .494 (n=5) | Jak2 |
| 1±.816 (n=5) | 1.306±.561 (n=5) | 2.603±.372 (n=5) | 4.107±.372 (n=5) | Stat6 |
| 1±.308 (n=5) | 1.020±.623 (n=5) | 2.186±.893 (n=5) | 3.585± .673 (n=5) | Muc5b |
| 1±.750 (n=5) | 1.448± .728 (n=5) | 4.768±.852 (n=5) | 7.160±.910 (n=5) | IFN-B |

## Slide 12
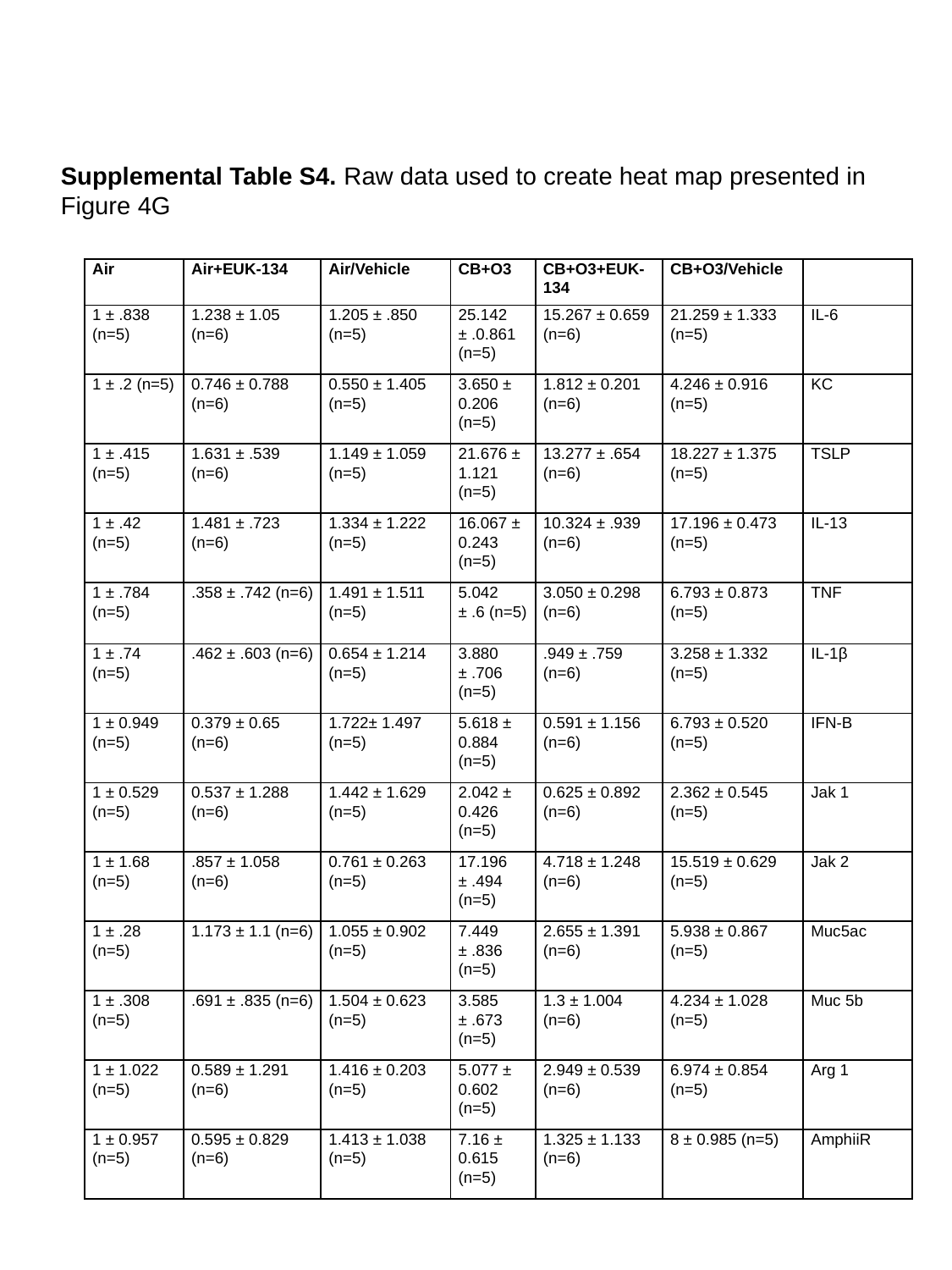

Supplemental Table S4. Raw data used to create heat map presented in Figure 4G
| Air | Air+EUK-134 | Air/Vehicle | CB+O3 | CB+O3+EUK-134 | CB+O3/Vehicle | |
| --- | --- | --- | --- | --- | --- | --- |
| 1 ± .838 (n=5) | 1.238 ± 1.05 (n=6) | 1.205 ± .850 (n=5) | 25.142 ± .0.861 (n=5) | 15.267 ± 0.659 (n=6) | 21.259 ± 1.333 (n=5) | IL-6 |
| 1 ± .2 (n=5) | 0.746 ± 0.788 (n=6) | 0.550 ± 1.405 (n=5) | 3.650 ± 0.206 (n=5) | 1.812 ± 0.201 (n=6) | 4.246 ± 0.916 (n=5) | KC |
| 1 ± .415 (n=5) | 1.631 ± .539 (n=6) | 1.149 ± 1.059 (n=5) | 21.676 ± 1.121 (n=5) | 13.277 ± .654 (n=6) | 18.227 ± 1.375 (n=5) | TSLP |
| 1 ± .42 (n=5) | 1.481 ± .723 (n=6) | 1.334 ± 1.222 (n=5) | 16.067 ± 0.243 (n=5) | 10.324 ± .939 (n=6) | 17.196 ± 0.473 (n=5) | IL-13 |
| 1 ± .784 (n=5) | .358 ± .742 (n=6) | 1.491 ± 1.511 (n=5) | 5.042 ± .6 (n=5) | 3.050 ± 0.298 (n=6) | 6.793 ± 0.873 (n=5) | TNF |
| 1 ± .74 (n=5) | .462 ± .603 (n=6) | 0.654 ± 1.214 (n=5) | 3.880 ± .706 (n=5) | .949 ± .759 (n=6) | 3.258 ± 1.332 (n=5) | IL-1β |
| 1 ± 0.949 (n=5) | 0.379 ± 0.65 (n=6) | 1.722± 1.497 (n=5) | 5.618 ± 0.884 (n=5) | 0.591 ± 1.156 (n=6) | 6.793 ± 0.520 (n=5) | IFN-B |
| 1 ± 0.529 (n=5) | 0.537 ± 1.288 (n=6) | 1.442 ± 1.629 (n=5) | 2.042 ± 0.426 (n=5) | 0.625 ± 0.892 (n=6) | 2.362 ± 0.545 (n=5) | Jak 1 |
| 1 ± 1.68 (n=5) | .857 ± 1.058 (n=6) | 0.761 ± 0.263 (n=5) | 17.196 ± .494 (n=5) | 4.718 ± 1.248 (n=6) | 15.519 ± 0.629 (n=5) | Jak 2 |
| 1 ± .28 (n=5) | 1.173 ± 1.1 (n=6) | 1.055 ± 0.902 (n=5) | 7.449 ± .836 (n=5) | 2.655 ± 1.391 (n=6) | 5.938 ± 0.867 (n=5) | Muc5ac |
| 1 ± .308 (n=5) | .691 ± .835 (n=6) | 1.504 ± 0.623 (n=5) | 3.585 ± .673 (n=5) | 1.3 ± 1.004 (n=6) | 4.234 ± 1.028 (n=5) | Muc 5b |
| 1 ± 1.022 (n=5) | 0.589 ± 1.291 (n=6) | 1.416 ± 0.203 (n=5) | 5.077 ± 0.602 (n=5) | 2.949 ± 0.539 (n=6) | 6.974 ± 0.854 (n=5) | Arg 1 |
| 1 ± 0.957 (n=5) | 0.595 ± 0.829 (n=6) | 1.413 ± 1.038 (n=5) | 7.16 ± 0.615 (n=5) | 1.325 ± 1.133 (n=6) | 8 ± 0.985 (n=5) | AmphiiR |

## Slide 13
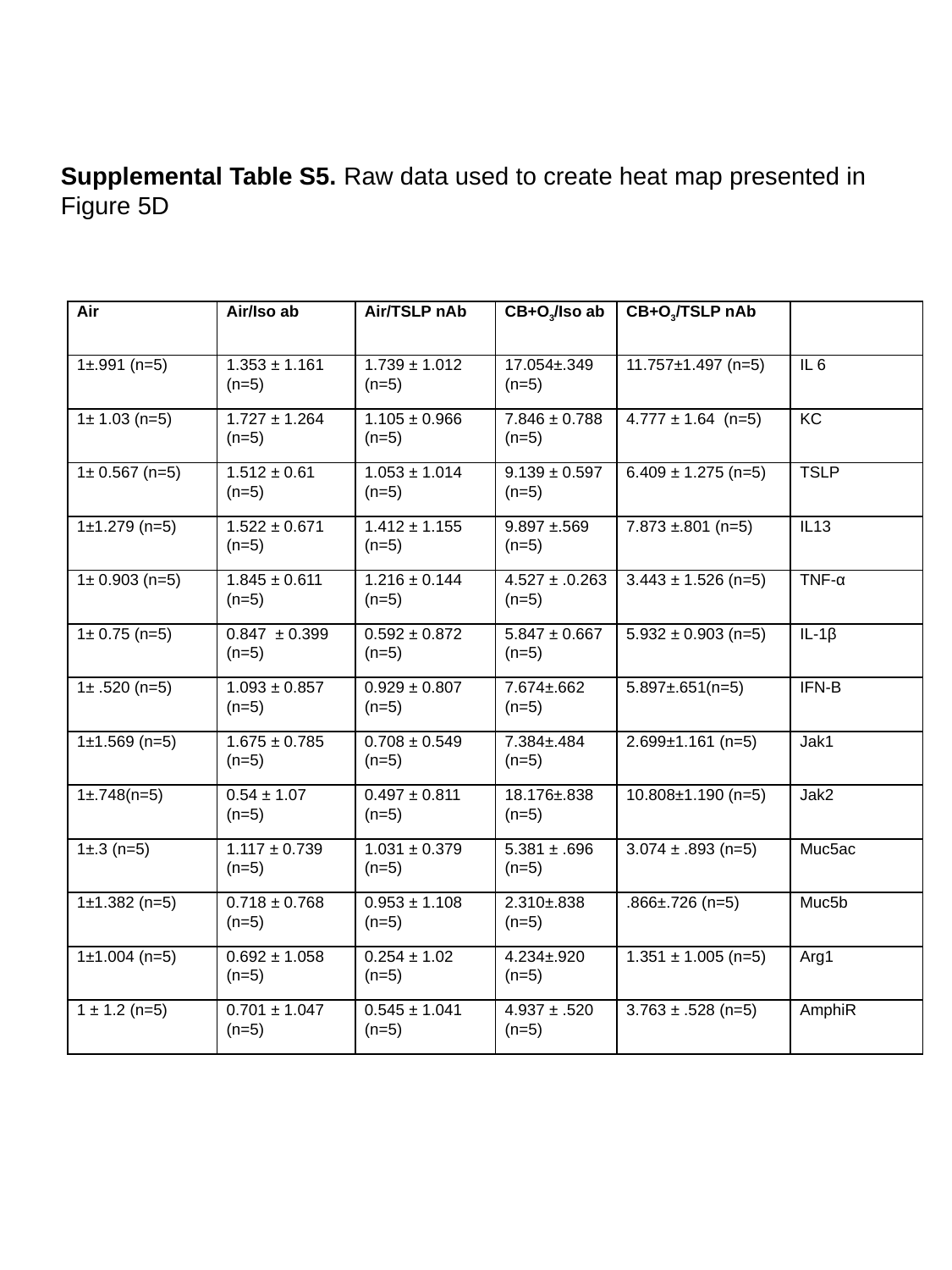

Supplemental Table S5. Raw data used to create heat map presented in Figure 5D
| Air | Air/Iso ab | Air/TSLP nAb | CB+O3/Iso ab | CB+O3/TSLP nAb | |
| --- | --- | --- | --- | --- | --- |
| 1±.991 (n=5) | 1.353 ± 1.161 (n=5) | 1.739 ± 1.012 (n=5) | 17.054±.349 (n=5) | 11.757±1.497 (n=5) | IL 6 |
| 1± 1.03 (n=5) | 1.727 ± 1.264 (n=5) | 1.105 ± 0.966 (n=5) | 7.846 ± 0.788 (n=5) | 4.777 ± 1.64 (n=5) | KC |
| 1± 0.567 (n=5) | 1.512 ± 0.61 (n=5) | 1.053 ± 1.014 (n=5) | 9.139 ± 0.597 (n=5) | 6.409 ± 1.275 (n=5) | TSLP |
| 1±1.279 (n=5) | 1.522 ± 0.671 (n=5) | 1.412 ± 1.155 (n=5) | 9.897 ±.569 (n=5) | 7.873 ±.801 (n=5) | IL13 |
| 1± 0.903 (n=5) | 1.845 ± 0.611 (n=5) | 1.216 ± 0.144 (n=5) | 4.527 ± .0.263 (n=5) | 3.443 ± 1.526 (n=5) | TNF-α |
| 1± 0.75 (n=5) | 0.847 ± 0.399 (n=5) | 0.592 ± 0.872 (n=5) | 5.847 ± 0.667 (n=5) | 5.932 ± 0.903 (n=5) | IL-1β |
| 1± .520 (n=5) | 1.093 ± 0.857 (n=5) | 0.929 ± 0.807 (n=5) | 7.674±.662 (n=5) | 5.897±.651(n=5) | IFN-B |
| 1±1.569 (n=5) | 1.675 ± 0.785 (n=5) | 0.708 ± 0.549 (n=5) | 7.384±.484 (n=5) | 2.699±1.161 (n=5) | Jak1 |
| 1±.748(n=5) | 0.54 ± 1.07 (n=5) | 0.497 ± 0.811 (n=5) | 18.176±.838 (n=5) | 10.808±1.190 (n=5) | Jak2 |
| 1±.3 (n=5) | 1.117 ± 0.739 (n=5) | 1.031 ± 0.379 (n=5) | 5.381 ± .696 (n=5) | 3.074 ± .893 (n=5) | Muc5ac |
| 1±1.382 (n=5) | 0.718 ± 0.768 (n=5) | 0.953 ± 1.108 (n=5) | 2.310±.838 (n=5) | .866±.726 (n=5) | Muc5b |
| 1±1.004 (n=5) | 0.692 ± 1.058 (n=5) | 0.254 ± 1.02 (n=5) | 4.234±.920 (n=5) | 1.351 ± 1.005 (n=5) | Arg1 |
| 1 ± 1.2 (n=5) | 0.701 ± 1.047 (n=5) | 0.545 ± 1.041 (n=5) | 4.937 ± .520 (n=5) | 3.763 ± .528 (n=5) | AmphiR |
